# LncRNA FENDRR with m6A RNA methylation regulates hypoxia-induced pulmonary artery endothelial cell pyroptosis by mediating DRP1 DNA methylation

**DOI:** 10.1186/s10020-022-00551-z

**Published:** 2022-10-25

**Authors:** Xiaoying Wang, Qian Li, Siyu He, June Bai, Cui Ma, Lixin Zhang, Xiaoyu Guan, Hao Yuan, Yiying Li, Xiangrui Zhu, Jian Mei, Feng Gao, Daling Zhu

**Affiliations:** 1 Central Laboratory of Harbin Medical University (Daqing), Daqing, 163319 People’s Republic of China; 2 College of Pharmacy, Harbin Medical University, Harbin, 150081 People’s Republic of China; 3 Key Laboratory of Cardiovascular Medicine Research, Ministry of Education, Harbin Medical University, Harbin, 150081 People’s Republic of China; 4 College of Medical Laboratory Science and Technology, Harbin Medical University (Daqing), Daqing, 163319 People’s Republic of China; 5 College of Dental Medicine-Illinois, Midwestern University, Downers Grove, IL 60515 USA; 6 College of Pharmacy, Harbin Medical University (Daqing), Xinyang Road, Daqing, 163319 Heilongjiang People’s Republic of China

**Keywords:** lncRNA FENDRR, Pyroptosis, m6A RNA methylation, Dynamin-related protein 1, Pulmonary artery endothelial cells

## Abstract

**Background:**

Pyroptosis is a form of programmed cell death involved in the pathophysiological progression of hypoxic pulmonary hypertension (HPH). Emerging evidence suggests that N6-methyladenosine (m6A)-modified transcripts of long noncoding RNAs (lncRNAs) are important regulators that participate in many diseases. However, whether m6A modified transcripts of lncRNAs can regulate pyroptosis in HPH progression remains unexplored.

**Methods:**

The expression levels of FENDRR in hypoxic pulmonary artery endothelial cells (HPAECs) were detected by using quantitative real-time polymerase chain reaction (qRT-PCR) and fluorescence in situ hybridization (FISH). Western blot, Lactate dehydrogenase (LDH) release assay, Annexin V-FITC/PI double staining, Hoechst 33342/PI fluorescence staining and Caspase-1 activity assay were used to detect the role of FENDRR in HPAEC pyroptosis. The relationship between FENDRR and dynamin-related protein 1 (DRP1) was explored using bioinformatics analysis, Chromatin Isolation by RNA Purification (CHIRP), Electrophoretic mobility shift assay (EMSA) and Methylation-Specific PCR (MSP) assays. RNA immunoprecipitation (RIP) and m6A dot blot were used to detect the m6A modification levels of FENDRR. A hypoxia-induced mouse model of pulmonary hypertension (PH) was used to test preventive effect of conserved fragment TFO2 of FENDRR.

**Results:**

We found that FENDRR was significantly downregulated in the nucleus of hypoxic HPAECs. FENDRR overexpression inhibited hypoxia-induced HPAEC pyroptosis. Additionally, DRP1 is a downstream target gene of FENDRR, and FENDRR formed an RNA–DNA triplex with the promoter of DRP1, which led to an increase in DRP1 promoter methylation that decreased the transcriptional level of DRP1. Notably, we illustrated that the m6A reader YTHDC1 plays an important role in m6A-modified FENDRR degradation. Additionally, conserved fragment TFO2 of FENDEE overexpression prevented HPH in vivo.

**Conclusion:**

In summary, our results demonstrated that m6A-induced decay of FENDRR promotes HPAEC pyroptosis by regulating DRP1 promoter methylation and thereby provides a novel potential target for HPH therapy.

**Supplementary Information:**

The online version contains supplementary material available at 10.1186/s10020-022-00551-z.

## Introduction

Hypoxic pulmonary hypertension (HPH) is a serious cardiovascular disease characterized by functional and structural changes of in pulmonary vasculature, which leads to increased pulmonary vascular resistance and remodeling, right ventricular hypertrophy, and finally death (Humbert 2019; McGoon et al. 2014; Thompson and Lawrie 2017). Studies have shown that endothelial cells (ECs) are the direct targets of hypoxia and are involved in the pathogenesis process of HPH leading to cell hyperproliferation, inhibition of apoptosis and plexiform intima injuries (Ranchoux et al. 2018; Masri et al. 2007). Therefore, dysfunctional ECs are important players in the imbalance of pulmonary vascular homeostasis and the pathogenesis of HPH.

Pyroptosis, a novel form of proinflammatory programmed cell death, is mainly dependent on the activation of caspase-1 or caspase-11 in inflammasomes (Shi et al. 2017; Bergsbaken et al. 2009). Studies have demonstrated that pyroptosis plays an important role in the development of infectious diseases, neurological diseases, atherosclerosis, acute and chronic liver diseases and immune system deficiency diseases (Man et al. 2017; McKenzie et al. 2020; Hoseini et al. 2018; Luan and Ju 2018; Mistry and Kaplan 2017). A recent study provided evidence that pyroptosis is involved in the inflammatory process of human pulmonary artery smooth muscle cells (HPASMCs) in HPH (Zhang et al. 2020). However, the role of pyroptosis in HPAEC, and its potential relationship with HPH as well as the underlying mechanisms are unclear.

Long noncoding RNAs (lncRNAs) are a large class of RNA molecules ranging in length from 200 to 100,000 nt and located in the nucleus or cytoplasm (Ponting et al. 2009). A large body of evidence has demonstrated that lncRNAs are engaged in various diseases, including HPH. For example, lncRNA-MEG3 sequesters miR-328-3p, leading to increased expression of IGF1R (Type 1 insulin-like growth factor receptor) and regulating the development of HPH (Xing et al. 2019). However, the reported lncRNAs in HPH are all focused on the cytoplasm of PASMCs, and the specific lncRNAs located in the nucleus, especially in PAECs involved in HPH progression and their related regulatory mechanism, remain largely unknown.

LncRNA FOXF1-AS1, also known as FENDRR, with its gene 3099 nt in length, is involved in some types of cancers, including cervical cancer, lung cancer and breast cancer. FENDRR could inhibit cervical cancer proliferation and invasion by targeting miR-15a/b-5p and regulating tubulin alpha1A expression (Zhu et al. 2020). FENDRR suppressed the progression of nonsmall cell lung cancer by regulating the miR-761/TIMP2 (tissue inhibitor of metalloproteinase 2) axis (Zhang et al. 2019). Because HPH has cancer-like phenotypes, such as hyperproliferation and apoptosis resistance, it is possible that FENDRR is involved in HPH pathogenesis.

N6-methyladenosine (m6A) RNA modification has been identified to regulate the expression of lncRNAs (Chen et al. 2020; He et al. 2020a).It is one of the most common epitranscriptomic modifications in eukaryotic RNAs and is regulated by m6A "writer" proteins (METTL3, METTL14), "eraser" proteins (FTO, ALKBH5) and "reader" proteins (YTHDC1-3, YTHDF1-3) (Zaccara et al. 2019; Wu et al. 2017). Recent studies have shown that m6A RNA modification plays an important role in all aspects of lncRNA metabolism by regulating the splicing, stability, translocation and translation of transcripts (Dai et al. 2018; Pan 2013). More importantly, m6A modification is reported to be important in the progression of multiple diseases (Jiang et al. 2021a). Although the roles of FENDRR in cancer have been elucidated, the specific mechanism of epigenetic modification regulation in FENDRR remains poorly understood.

In the current study, we identified that FENDRR was downregulated in hypoxic HPAEC, and involved in hypoxia-induced pyroptosis of HPAECs. Mechanistically, YTHDC1-mediated m6A modification induced the downregulation of FENDRR, which subsequently promoted hypoxia-induced HPAEC pyroptosis by decreasing the formation of an RNA–DNA triplex in the promoter region of dynamin-related protein 1 (DRP1) to inhibit promoter DNA methylation. Our results reveal a novel regulatory mechanism for hypoxia-induced HPAEC pyroptosis and provide a potential target with therapeutic implications in HPH.

## Materials and methods

### Animals and lung tissue preparation

Healthy male C57BL/6J mice with a mean weight of 30 g were obtained from the Experimental Animal Center of Harbin Medical University (Harbin, China). To confirm the role of the functional fragment TFO2 of FENDRR (464–516) in HPH, The TFO2 sequence of FENDRR cloning construction and serotype 5 adenovirus-associated virus (AAV 5) packaging experiment were constructed by HANBIO (Shanghai, China). An aliquot of the vector at 10^11^ genome equivalents was prepared in 20–30 μL of HBSS and isoflurane anesthesia followed by nasal drops. Mice were randomly divided into five groups as follows: normoxic environment plus control vector group (NOR + NC, n = 20), hypoxic environment plus control vector group (HYP + NC, n = 10), hypoxic environment plus FENDRR TFO2 adenovirus group (HYP + FENDRR TFO2, n = 10), normoxic environment plus FENDRR TFO2 adenovirus group (NOR + FENDRR TFO2, n = 10). Seven days later, mice were assigned to normoxia (Fi,O_2_ 0.21) and hypoxia (Fi,O_2_ 0.10) for seven days as previously described (Zhu et al. 2003). The mice were administered nontargeted control vector or FENDRR TFO2 adenovirus intranasally again and were assigned to normoxia (Fi,O_2_ 0.21) and hypoxia (Fi,O_2_ 0.10) for 14 days. All mice were anaesthetized through an intraperitoneal injection of avertin (200 mg/kg i.p., Sigma-Aldrich, St Louis, USA). For the right ventricular hypertrophy index (ratio of right ventricular free wall weight over the sum of septum plus left ventricular free wall weight: RV/(LV + Sep) calculation, hearts were excised and atria were removed. The RV free wall was dissected, and each cham-ber was weighed.

### Echocardiography and right ventricular systolic pressure (RVSP) measurements

The right ventricular systolic pressure (RVSP) and echocardiography were measured as previously described (Liu et al. 2020a). The right ventricular systolic pressure (RVSP) was measured with PowerLab monitoring equipment (AD Instruments, Colorado Springs, CO). A 1.2 French Pressure Catheter (Scisense Inc, USA) was inserted into the superior vena cava and finally into the right ventricular vein, and the RVSP was continuously recorded for 20–40 min. Mice were subjected to echocardiography using a Vevo2100 imaging system (VisualSonics Inc., Toronto, Ontario, Canada), pulmonary artery velocity time integral (PAVTI), pulmonary artery acceleration time (PAAT) and left ventricular ejection fraction (LVEF) were obtained from stable images.

### Morphometric analysis

Hematoxylin and eosin staining (HE staining) was performed according to the manufacturer’s instructions. In brief, lung tissues of mice were immersed in 4% paraformaldehyde for 48 h. Next, the fixed lung tissues were dehydrated, cleared and embedded in paraffin wax. The lung tissue volume of each block was sampled with equal probability. The paraffin blocks were cut into 5-μm-thick sections and stained with hematoxylin and eosin (HE). In situ hybridization was performed with kits following the manufacturer’s instructions (Boster, Wuhan, China). Digoxigenin-labeled DNA probes complementary to TFO2 of FENDRR were generated using random primer labeling. For each slice stained, 6 high power fields were randomly selected for analysis. The total wall thickness and positive staining area in the vascular walls were quantified by using a color-recognition algorithm in Image-Pro Plus 6.0 software.

### Cell culture

HPAECs used in the experiment were purchased from ScienCell Research Laboratories (CA, USA). HPAECs were maintained in endothelial cell medium (ScienCell, 1001, CA, USA) containing 15% fetal bovine serum and 1% penicillin streptomycin at 37 °C, 5% CO_2_, and 100% relative humidity. Cells under hypoxic conditions were incubated in a Tri-Gas Incubator (Heal Force) with a water-saturated atmosphere comprising 3% O_2_, 5% CO_2_ and 91% N_2_ for 24 h.

### Fluorescent in situ hybridization (FISH)

Fluorescence-conjugated FENDRR probes were synthesized by RuiBo (Guangzhou, China). FISH experiments were performed using a Fluorescent In Situ Kit (RuiBo Biology, Guanzhou, China) following the manufacturer’s instructions. Briefly, HPAECs were cultured on coverslips and then grown to approximately 60%. After being treated with agents according to the different experimental groups, cells were washed with 1 × PBS, fixed with 4% paraformaldehyde, and permeabilized with 0.3% Triton X-100. Then, the cells were blocked with prehybridization solution at 37 °C for 1 h and incubated with hybridization solution containing FENDRR, 18S and U6 probes overnight at 37 °C in the dark. Finally, 4′,6-diamidino-2-phenylindole (DAPI) was added to stain the nuclei at 37 °C for 10 min. Images were captured with a living cell workstation (AF6000; Leica, Germany).

### Isolation of cytoplasmic and nuclear RNAs

Cytoplasmic and nuclear RNAs were isolated and purified using a Norgen’s Cytoplasmic & Nuclear RNA Purification Kit (Thorold, ON, Canada) following the manufacturer’s instructions. In brief, 1 × 10^6^ HPAECs were lysed with ice-cold lysis buffer, and cytoplasmic RNA and nuclear RNA were bound to the column. Finally, the mixture was separated for RNA elution analysis.

### Western blot analysis

Protein samples were extracted from HPAECs by using ice-cold lysis buffer and then centrifuged at 13,500 rpm for 15 min at 4 ℃. After centrifugation, the protein concentrations were determined using a Bio-Rad protein assay kit (Bio-Rad Laboratories, Inc., Berkeley, CA, USA). Protein samples (30 µg) were fractionated on 12% SDS-PAGE gels, transferred onto nitrocellulose membranes, and subsequently blocked with 5% nonfat milk at room temperature for 1 h. The membranes were incubated with specific antibodies against NLRP3 (2 µg/mL, bs-10021R, Bioss, Beijing, China), Caspase-1 (1 µg/mL, 22915-1-AP, Proteintech, IL, USA), pro-Caspase-1 (1 µg/mL, ab179515, Abcam, MA, USA), IL-1β (1 µg/mL, 16806-1-AP, Proteintech, IL, USA), DRP1 (1 µg/mL, ab184247, Abcam, MA, USA), YTHDC1 (2 µg/mL, 14392-1-AP, Proteintech, IL, USA), FTO (2 µg/mL, bs-7056R, Bioss, Beijing, China). Bands were sequentially incubated with horseradish peroxidase-labeled secondary antibodies at room temperature for 1 h and enhanced chemiluminescent reagent imaging.

### Quantitative RT–PCR

Total RNA was extracted from HPAECs using TRIzol reagent (Invitrogen, Carlsbad, CA) according to the manufacturer’s instructions. The concentration and purity of all samples were measured via a NanoDrop 2000 (Thermo Scientific, Wilmington, USA), and cDNA was synthesized with the use of the Superscript first-strand complementary DNA synthesis kit (Invitrogen). Finally, the products were quantified using SYBR Green real-time PCR (Toyobo, Japan) in a Roche LightCycler 480II instrument. The nucleotide sequences of the primers are shown in Table 1.

**Table 1:** All primers and probes sequences

RT-PCR primers		
Name	Position	Sequences (5′-3′)
Hum-FENDRR	Forward primer	CTCCCGTGGAAGCCATTTCT
	Reverse primer	CCTCTGGCTGCGTTTTTCAC
Hum-DRP1	Forward primer	GTTTTTCCATGTAGCAGGGTCA
	Reverse primer	ACCTGCTTCCCAGAGGTACT
Mus-Caspase-1	Forward primer	AGGCACGGGACCTATGTGAT
	Reverse primer	AGGGCAAAACTTGAGGGTCC
Mus-NLRP3	Forward primer	TGGGTTCTGGTCAGACACGAG
	Reverse primer	GGCGGGTAATCTTCCAAATGC
Mus-IL-1β	Forward primer	GCCACCTTTTGACAGTGATGAG
	Reverse primer	AAGGTCCACGGGAAAGACAC
Hum-β-actin	Forward primer	CTCACCATGGATGATGATATCGC
	Reverse primer	CACATAGGAATCCTTCTGACCCA
Mus-β-actin	Forward primer	TGCTTCTAGGCGGACTGTTAC
	Reverse primer	AACCAACTGCTGTCGCCTT
DRP1 promoter	Forward primer	CCTCTCCTCACCTGCTTTAATTC
	Reverse primer	GTATCCCTCCTCTACTCCAAACC

MSP primers		
Name	Position	Sequences (5′-3′)
DRP1 CpG methylation	Forward primer	TAATTTTAATATTTTGGGAGGTCGA
	Reverse primer	CCAAACTAAAATACAATAACGCGAT
DRP1 CpG unmethylation	Forward primer	TAATTTTAATATTTTGGGAGGTTGA
	Reverse primer	CCAAACTAAAATACAATAACACAAT

Other primers		
Name	Position	Sequences (5′-3′)
DRP1 siRNA	Sense	GCCUUAACACUAUUGACAUTT
	Antisense	AUGUCAAUAGUGUUAAGGCTT
YTHDC1 siRNA1	Sense	GGAGAAAGAUGGAGAACUUTT
	Antisense	AAGUUCUCCAUCUUUCUCCTT
YTHDC1 siRNA2	Sense	GCUCUGCAUCAGAGUCAUATT
	Antisense	UAUGACUCUGAUGCAGAGCTT
YTHDC1 siRNA3	Sense	GCAAGGAGUGUUAUCUUAATT
	Antisense	UUAAGAUAACACUCCUUGCTT
Negative control	Sense	UUCUCCGAACGUGUCACGUTT
	Antisense	ACGUGACACGUUCGGAGAATT

CHIRP probes		
Negative control	Single strand	UUGUACUCACAAAAGUACUG
FENDRR TFO1	Single strand	CGCGCACCUGGCUGCCAGCCCGCAGGGGGCUCGCACGCAGACCUG
FENDRR TFO2	Single strand	AGAAGAAAAAACACAAAAUACCCAACCACAGAUCCUCAAAAAUAU

### Plasmid vector constructs and transfection

For the overexpression assay, FENDRR and DRP1 plasmids were constructed using the vector GV219, and empty vector alone was used as a negative control (GeneChem, Shanghai, China). HPAECs were transfected with 3 μg of plasmids using Lipofectamine 2000 reagent following the manufacturer’s instructions. Then, 4–6 h after transfection, the cells were switched to 5% serum-containing medium and cultured under normoxic or hypoxic growth conditions for another 24 h.

### siRNA, ASO design and transfection

The expression of genes was silenced by transfecting HPAECs with small interfering RNAs (siRNAs) or antisense oligonucleotides (ASOs), which were designed and synthesized by GenePharma (Shanghai, China) and RiboBio (Guangzhou, China). HPAECs were transfected with 2 μg of siRNAs or ASO using X-tremeGene siRNA transfection reagents following the manufacturer’s instructions. Six hours after transfection, the cells were switched to 5% serum-containing medium and cultured under normoxic or hypoxic growth conditions for another 24 h. The detailed siRNA sequences are shown in Table 1.

### Hoechst 33342/PI fluorescent staining

HPAECs were cultured on coverslips until the cell confluence reached 80%. The cells were treated with different agents according to the different experimental groups. Afterward, the cells were stained with 6 μL of Hoechst 33342 solution and 6 μL of PI (propidium iodide) at 4 °C in the dark for 20 min. Images were captured with a living cell workstation (AF6000; Leica, Germany).

### LDH release assay

HPAECs were plated into 96-well plates at a density of 5000 cells/well, and then cells were treated with different agents according to the different experimental groups. After 24 h of hypoxia, LDH was measured according to the LDH Release Assay Kit instructions (Beyotime Biotechnology, Shanghai, China). Finally, absorbance at 490 nm was recorded.

### Cell and tissue immunofluorescence

HPAECs were cultured on coverslips in 12-well plates and then treated with different agents according to the different experimental groups. Prepared cells were washed three times with 1 × PBS and were fixed with 4% paraformaldehyde at 4 °C for 15 min. Then, the cell membrane was permeabilized with 0.3% Triton X-100 for 30 min and blocked with 5% bovine serum for 30 min at room temperature. After that, the cells were incubated with DRP1 (10 µg/mL, ab184247, Abcam, MA, USA), Caspase-1 (6 µg/mL, 22915-1-AP, Proteintech, IL, USA), NLRP3 (10 µg/mL, bs-10021R, Bioss, Beijing, China) and CD31 (5 µg/mL, ab9498 Abcam, MA, USA) antibodies in PBS at 4 °C overnight. After washing three times with 1 × PBS, the cells were subsequently incubated with Cy3-conjugated goat anti-rabbit (A0516, Beyotime, Shanghai, China) and FITC-conjugated goat anti-mouse (A0568, Beyotime, Shanghai, China) antibodies for 2 h at 37 °C in the dark. Cells were then washed with 1 × PBS, DAPI (C1002, Beyotime, Shanghai, China) was added to stain the nuclei at 37 °C for 10 min. Finally, the coverslips were mounted with anti-fade mounting medium (P0126, Beyotime, Shanghai, China) and captured with a living cell workstation. The frozen sections of mouse lung tissues were performed in the same manner.

### RNA immunoprecipitation (RIP)

The RNA immunoprecipitation assay was performed by using an RNA Immunoprecipitation (RIP) Kit (Bes5101, BersinBio, Guangzhou, China) following the manufacturer’s instructions. Briefly, 1 × 10^7^ HPAECs were lysed with RIP lysis buffer. After removing DNA, 20 µL of protein A/G bead-conjugated anti-YTHDC1 antibodies (3 µg, 14392-1-AP, Proteintech, IL, USA) and IgG were added to the samples and incubated overnight at 4 °C. After extracting RNA, the expression of FENDRR was detected by qRT-PCR. For m6A-RNA immunoprecipitation (Me-RIP), an anti-m6A antibody (4 µg A-1801, Epigentek Group Inc., Farmingdale, NY) was used. FENDRR extracted from cell lysates was used to measure the m6A-methylated level of FENDRR.

### Chromatin isolation by RNA purification (CHIRP)

The interaction between FENDRR and the promoter of DRP1 was determined using chromatin isolation by RNA purification assays according to the instruction manual of the Chromatin Isolation by RNA Purification (ChIRP) Kit (Bes5104, BersinBio, Guangzhou, China). Briefly, 4 × 10^7^ HPAECs were collected and crosslinked with 1% formaldehyde for 20 min at room temperature. Crosslinking was stopped by adding glycine to the cell suspension for 5 min. Then, the cells were lysed with CHIRP lysis buffer and sonicated to obtain DNA fragments of approximately 100–500 bp. Samples were precleared and incubated with FENDRR probes (TFO1, TFO2) at 37 °C for 180 min. Finally, DNA was isolated and subjected to qPCR. The specific biotinylated probes TFO1 and TFO2 were synthesized by GenePharma (Shanghai, China). The sequences are shown in Table 1.

### Dual-luciferase reporter assay

The DRP1 promoter fragment containing the FENDRR binding site was cloned into the GV238 plasmid expressing luciferase (Genepharma, Shanghai, China). HPAECs were cotransfected with the FENDRR expression plasmid and DRP1 plasmid expressing luciferase with Lipofectamine 2000 for 48 h. Then, the luciferase activities were measured by the dual-luciferase reporter assay system (Promega, USA).

### Electrophoretic mobility shift assay (EMSA)

The specific biotinylated probe containing the DRP1 TSS fragment and the transcribed FENDRR TFO fragment was synthesized by GenePharma (Shanghai, China). The electrophoretic mobility shift assay (EMSA) was performed by a Chemiluminescent EMSA Kit (GS009, Beyotime, Shanghai, China) following the manufacturer’s instructions. In brief, the biotinylated DRP1 TSS and the synthesized FENDRR TFO2 were reacted in 10 mL of binding reaction buffer at room temperature for 20 min. Then, the sample was added to a 4% nondenatured polyacrylamide gel for electrophoresis purposes and transferred onto a nylon membrane, followed by UV crosslinking at 245 wavelengths. The membrane was incubated with streptavidin-HRP conjugate and enhanced chemiluminescent reagent imaging.

### Methylation-specific PCR (MSP)

The methylation level of the DRP1 promoter region was detected by a GENMED Universal Gene Methylation Detection Kit (GENMED Scientifics INC.USA). In brief, a genomic DNA extraction kit (K0512, Thermo Scientific, USA) was used to extract genomic DNA. Then, 2 µg of DNA was transformed with GENMED reagents and subsequently subjected to PCR. The PCR programs were as follows: predenaturation at 95 °C for 2 min, 35 cycles of denaturation at 95 °C for 30 s, 57 °C for 90 s, and annealing at 72 °C for 30 s, with the last extension at 72 °C for 5 min. Finally, the PCR products were analyzed by 2% agarose gel electrophoresis and captured with a gel imaging system. The primers for methylation and unmethylation of the DRP1 promoter were shown in Table 1.

### Global RNA m6A quantification

The EpiQuik m6A RNA Methylation Quantification Kit (Colorimetric, P-9005, Epigentek Group Inc., Farmingdale, NY) was used to detect global m6A modifications in total HPAEC RNAs following the manufacturer’s instructions. Briefly, 2 µL of NC, 2 µL of PC and 200 ng of RNA were added into strip wells, and the solution was mixed. m6A were detected using capture and detection antibodies. The detected signal was enhanced and then quantified colorimetrically at a wavelength of 450 nm in a spectrophotometer.

### m6A dot blot assay

Two micrograms of total RNA were deposited on a nylon membrane (FFN10, Beyotime Biotechnology, Shanghai, China), and then the nylon membrane was crosslinked by UV for 3 min. Next, the nylon membrane was stained by using methylene blue. Subsequently, the nylon membrane was blocked for 1 h in blocking buffer, and the membrane was incubated with m6A antibody (1 µg/mL, A-1801, Epigentek Group Inc., Farmingdale, NY) at 4 ℃ overnight. The membrane was incubated with horseradish peroxidase-labeled secondary antibodies at room temperature for 1 h and enhanced chemiluminescent reagent imaging.

### Flow cytometry assay

Pyroptosis of HPAECs was detected by using an Annexin V-FITC Detection Kit (C1062S, Beyotime Biotechnology, Shanghai, China) according to the manufacturer’s instructions. HPAECs were collected and stained with annexin V-FITC and PI at room temperature for 20 min. Afterward, the samples were analyzed using a BD FACSCalibur Flow Cytometer (BD Biosciences, Bedford, MA).

### Correlation analysis

Total RNA samples were extracted from HPAECs of the normoxia group and hypoxia group, and reverse transcribed into cDNA. Then, real-time PCR was used to measure the mRNA expression levels of FENDRR and DRP1. The correlation of FENDRR and DRP1 was analyzed using the Pearson correlation test of GraphPad Prism 8.0, and P < 0.05 was considered significant.

### Caspase-1 activity assay

The caspase-1 activity was detected using a caspase-1 activity assay kit (C1102, Beyotime Biotechnology, Shanghai, China) according to the manufacturer’s instructions. Briefly, 2 × 10^6^ HPAECs were harvested and lysed on ice for 15 min, centrifuged at 16,000×*g* for 15 min, and the supernatant was mixed with synthetic tetrapeptide Ac-YVAD-pNA and incubated at 37 °C for overnight. Finally, absorbance at 405 nm was recorded. The concentrations of total proteins were measured by a Bradford assay kit (P0006, Beyotime Biotechnology, Shanghai, China) according to the manufacturer’s instructions. The caspase-1 activity was calculated by the standard curve of pNA.

### Bioinformatic analysis

To analyze FENDRR localization, the lncATLAS website (http://lncatlas.crg.eu/) was used. Secondary structure analysis of FENDRR was performed using the RNAfold web server (http://rna.tbi.univie.ac.at/cgibin/RNAWebSuite/RNAfold.cgi). The target proteins prediction of FENDRR was performed through AnnoLnc (http://annolnc.gao-lab.org/index.php) and RNAInter (https://www.rna-society.org/raid/). A Venn diagram was shown using Venny^2.1^ web server (https://bioinfogp.cnb.csic.es/tools/venny/index.html). KEGG and GO enrichment were analyzed with the DAVID website (https://david.ncifcrf.gov/). The FENDRR TFO sequence and DRP1 promoter TTS sequence were identified with LongTarget (http://lncrna.smu.edu.cn/show/DNATriplex). Space structure docking of FENDRR TFO2 and DRP1 TTS was performed using HNADOCK Server (http://huanglab.phys.hust.edu.cn/hnadock/). CpG islands in the DRP1 gene promoter region were analyzed using Methyl Primer Express (http://www.urogene.org/cgi-bin/methprimer/methprimer.cgi). The N6-methyladenosine (m6A) modification site of FENDRR was predicted using the SRAMP prediction server (http://www.cuilab.cn/sramp).

### Statistical analysis

Statistical analyses were performed using GraphPad Prism Software 8.0 (GraphPad Software Inc.). Data are expressed as mean ± SD. All expression values were checked for normal distribution before statistical. Student's t test was used to compare the data between two groups and one-way ANOVA with Tukey post hoc test was used to compare between multiple groups. For non-normally distributed data, we performed nonparametric analyses such as the Mann–Whitney U test for two groups or Kruskal–Wallis test followed by Dunn post-test for multiple groups. Results with 2-tailed of P < 0.05 were considered statistically significant.

## Results

### Hypoxia downregulates the expression of FENDRR in HPAECs

**Fig. 1:**
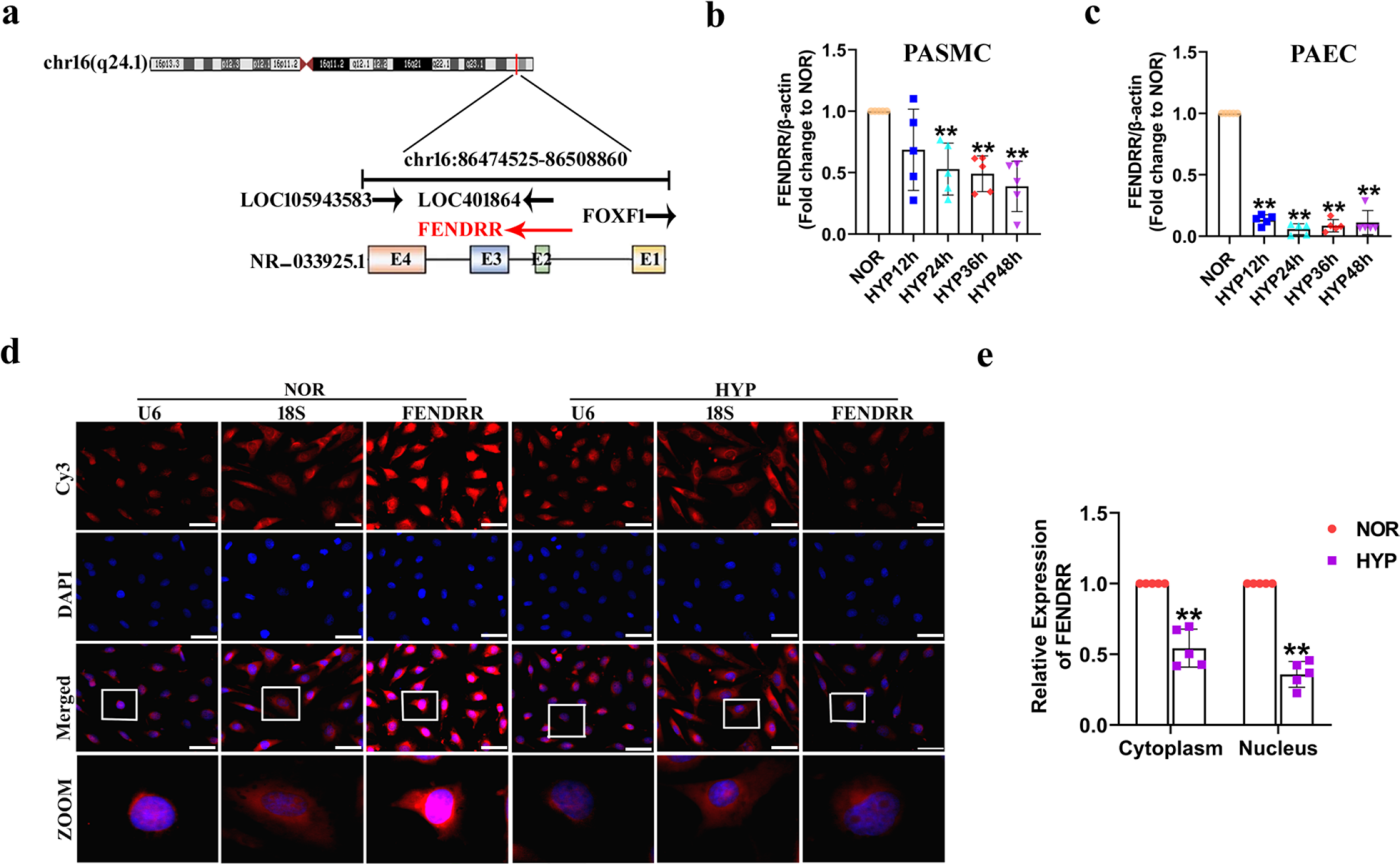
FENDRR expression is decreased under hypoxic conditions. **a** Genomic location of FENDRR. The arrows indicate the direction of transcription. **b** Expression of FENDRR quantified by qRT-PCR in human pulmonary artery smooth muscle cells endothelial cells (HPASMCs) (n = 5). **c** Expression of FENDRR quantified by qRT-PCR in human pulmonary artery endothelial cells (HPAECs) (n = 5). **d** HPAECs were cultured for 24 h under HYP conditions, and fluorescence in situ hybridization (FISH) assay was performed to detect FENDRR expression. U6 and 18S RNA were used as controls for localization of the nucleus and cytoplasm. Scale bar = 50 μm. **e** FENDRR expression in the nucleus and cytoplasm of HPAECs after exposure to HYP for 24 h (n = 5). Each datapoint in the figure represents a unique biological replicate. All values are presented as the mean ± SD. Statistical analysis was performed with Student’s t-test. NOR: normoxia; HYP: hypoxia. **P < 0.01 compared with NOR

In the NCBI browser, FENDRR was located on human chromosome 16:86474525–86508860 and was 3099 nt in length (Fig. 1a). For quantitative analysis of FENDRR, a specific primer was designed (Additional file 1: Fig. S1a). First, to investigate the significance of FENDRR in hypoxic HPASMCs and HPAECs, we examined the expression of FENDRR by qRT–PCR. The results showed that FENDRR was downregulated in a time-dependent manner in hypoxic HPASMCs and HPAECs, but it decreased 9.28-fold at 24 h in HPAECs than in HPASMCs (Fig. 1b and c). Then, we used the lncATLAS website to predict the subcellular localization of FENDRR, the results of which revealed that FENDRR was mainly localized in the nucleus (Additional file 1: Fig. S1b). The distribution of FENDRR mostly in the nucleus was further confirmed by fluorescence in situ hybridization (FISH) analysis (Fig. 1d). Additionally, cellular fractionation experiments showed that FENDRR was downregulated by 1.84-fold and 2.79-fold in both the cytoplasm and nucleus under hypoxic conditions (Fig. 1e). Finally, the RNAfold Web server was used to analyze the secondary structure of FENDRR, which proved its stability (Additional file 1: Fig. S1c). The above results demonstrated that the expression of FENDRR was downregulated in hypoxia-induced HPAECs and may be a key regulator involved in HPH.

### FENDRR inhibits HPAEC pyroptosis induced by hypoxia

**Fig. 2:**
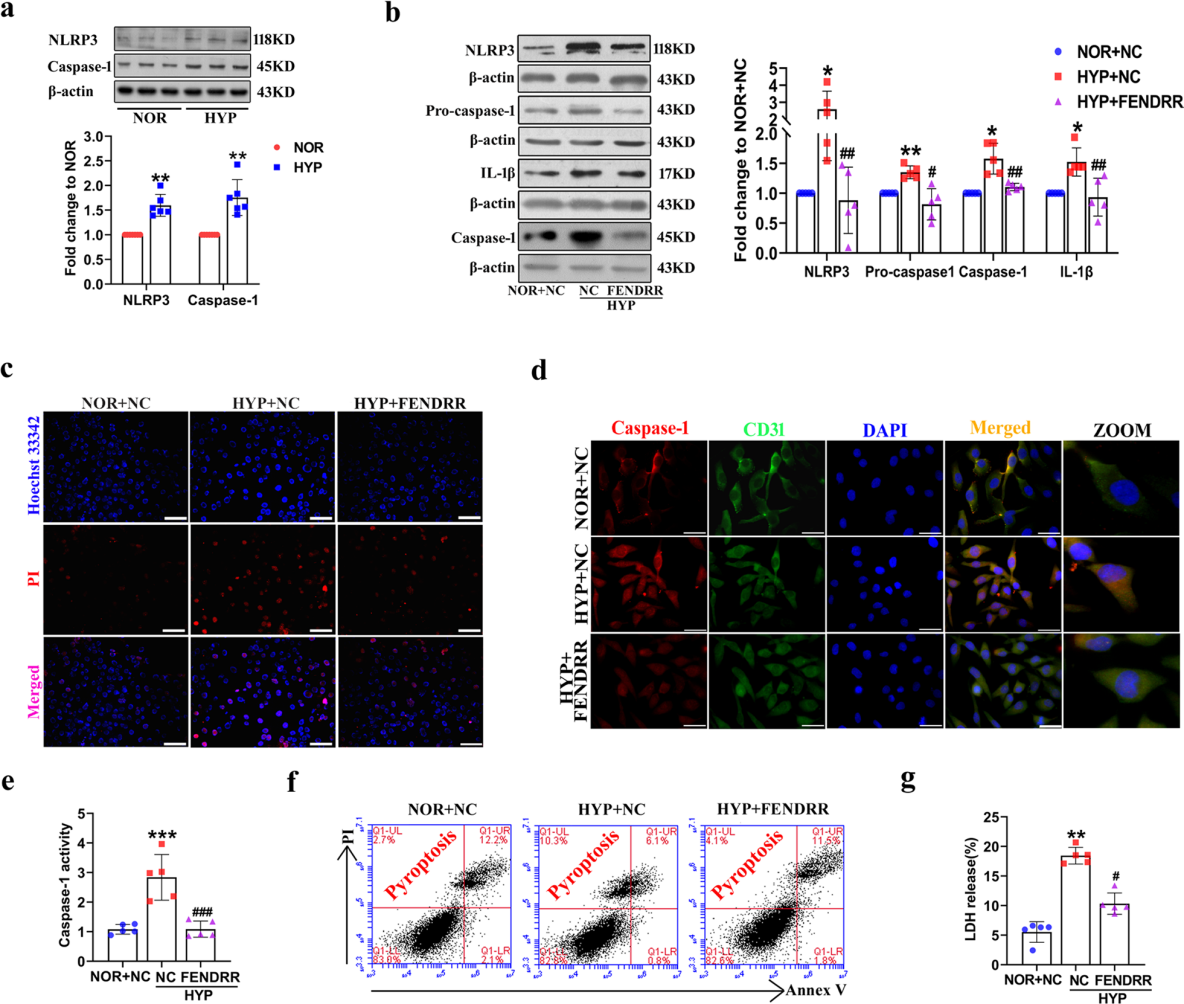
Overexpression FENDRR inhibits hypoxia-induced HPAECs pyroptosis. **a** The protein levels of NLRP3 and Caspase-1 were elevated after hypoxia exposure 24 h (n = 6). **b** Overexpression FENDRR reversed the increased protein levels of Caspase-1, NLRP3, Pro-caspase-1 and IL-1β induced by hypoxia in HPAECs (n = 5). **c** The positive PI (red) cells were detected in hypoxic HPAECs transfected with FENDRR overexpression plasmid. Scale bar = 50 μm. **d** Fluorescence staining of Caspase-1 in HPAECs transfected with FENDRR overexpression plasmid. HPAECs were stained for Caspase-1 (red) and CD31 (green), and DAPI (blue) was used for nuclear staining. Scale bar = 50 μm. **e** The activity of caspase-1 was examined using caspase-1 activity assay kit (n = 5). **f** The Flow cytometry assay was used to detect the population of positive-PI cells. HPAECs were treated with annexin V-FITC/propidium iodide (PI) double staining using quantitative fluorescence-activated cell sorting (FACS) analysis. **g** HPAECs were transfected with FENDRR overexpression plasmid under hypoxia and the LDH release was evaluated by LDH release kit (n = 5). Each datapoint in the figure represents a unique biological replicate. All values are presented as the mean ± SD. Statistical analysis was performed with one-way ANOVA or the Student’s t-test. NOR: normoxic; HYP: hypoxic; NC: negative control. *P < 0.05, **P < 0.01, ***P < 0.001 compared with NOR + NC. ^#^p < 0.05, ^##^p < 0.01, ^###^p < 0.001 compared with HYP + NC

Several studies have shown that hypoxia can induce PASMC pyroptosis (Jiang et al. 2021b; He et al. 2020b), but whether hypoxia affects PAECs pyroptosis is unclear. To confirm the role of hypoxia in pyroptosis of HPAECs, pyroptosis-related proteins were first assessed by Western blot. After 24 h of hypoxia, we found that NLRP3 and Caspase-1 were upregulated by 1.60-fold and 1.75-fold high in hypoxia HPAECs (Fig. 2a). Next, we evaluated the functional role of FENDRR in HPAEC pyroptosis under hypoxic conditions using the FENDRR overexpression plasmid. A sketch map for plasmid construction of FENDRR overexpression is shown in the supplement (Additional file 1: Fig. S2a). The overexpression efficiency was verified by qRT–PCR and FISH (Additional file 1: Fig. S2a and b). Hypoxia-induced upregulation of pyroptosis-related proteins expression was reversed by FENDRR overexpression in HPAECs (Fig. 2b). At the same time, hypoxia increased the positive PI staining, and the effect was inhibited by FENDRR overexpression under the same conditions (Fig. 2c). Immunofluorescence assays indicated that FENDRR overexpression attenuated Caspase-1 expression under hypoxic environments (Fig. 2d). Caspase-1 activity levels also were decreased by FENDRR overexpression in hypoxia HPAECs, the relative values are 1.08 ± 0.16, 2.84 ± 0.77, 1.09 ± 0.27 (Fig. 2e). In addition, FENDRR overexpression inhibited the pyroptotic cell death from 10.3% to 4.1% in HPAECs compared with hypoxic groups. (Fig. 2f). The LDH release assay results indicated that FENDRR overexpression attenuated the increased LDH activity from 18.4% to 10.3% in HPAECs exposed to hypoxia (Fig. 2g). Moreover, the results from western blot assay show that the expression of Caspase-4 and Caspase-11 (nonclassical pyroptosis pathway) was unaffected by FENDRR overexpression in hypoxia HPAECs (Additional file 1: Fig. S2c). These results demonstrate that FENDRR is an important participant in the regulation of hypoxia-induced HPAEC classical pyroptosis pathway.

### FENDRR knockdown enhances cell pyroptosis in HPAECs

To further reveal the effects of FENDRR in vitro, FENDRR was knocked down in the nucleus of HPAECs using antisense oligonucleotides (ASOs), and a sketch map of the antisense oligonucleotides (ASOs) of FENDRR is shown in Additional file 1: Fig. S3a. The transfection efficiency was verified by qRT–PCR and FISH (Additional file 1: Fig. S3a and b). FENDRR knockdown promoted expression of pyroptosis-related proteins by at least 1.5-fold and positive PI staining in HPAECs (Additional file 1: Fig. S3c and d). Moreover, pyroptotic cell death in HPAECs increased from 1.3% to 3.9% after transfection of FENDRR ASOs (Additional file 1: Fig. S3e). FENDRR knockdown in HPAECs increased the release of LDH activity from 7.3% to 16.7% and upregulated the fluorescence intensity of Caspase-1 (Additional file 1: Fig. S3f and g). Taken together, these results imply that FENDRR negatively regulates the pyroptosis of HPAECs.

### FENDRR regulates the expression of DRP1 in HPAECs

**Fig. 3:**
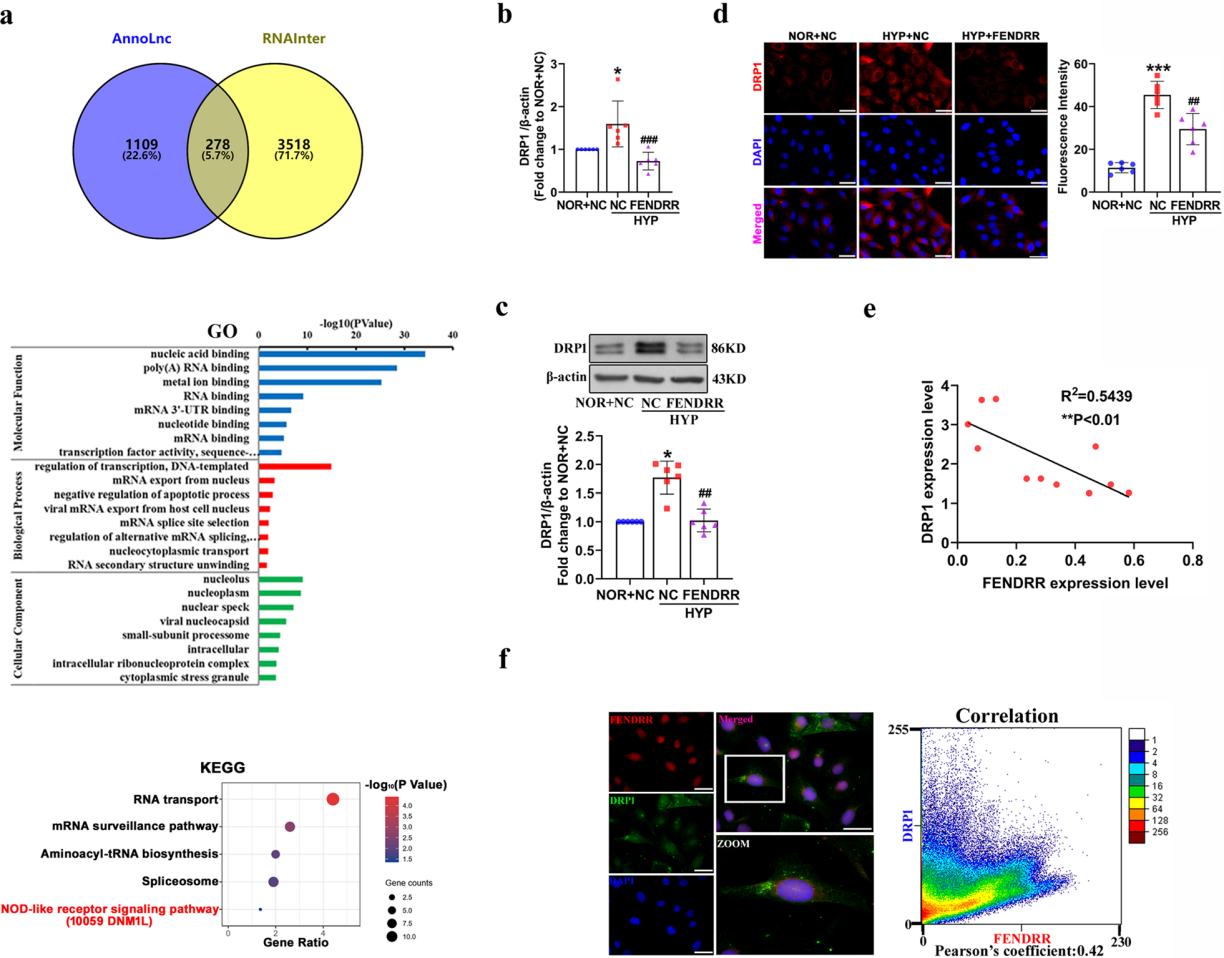
DRP1 is regulated by FENDRR in HPAECs. **a** AnnoLnc and RNAInter bioinformatics software predicted FENDRR target proteins, and Gene Ontology (GO) and Kyoto Gene and Genomic Encyclopedia (KEGG) analysis of 278 genes in intersection. **b** Expression of DRP1 quantified by qRT-PCR (n = 5). **c** Western blot was used to verify the expression of DRP1 (n = 5). **d** Fluorescence staining for DRP1. HPAECs were stained for DRP1 (red) and DAPI (blue) was used for nuclear staining. Scale bar = 50 μm. **e** Analysis of the correlation between FENDRR and DRP1 in HPAECs. **f** Colocalization of FENDRR and DRP1in HPAECs. Scale bars = 50 µm. FENDRR probes were labeled with Cy3 (red), DRP1 were stained with FITC (green) and nuclei were stained with DAPI (blue). Pearson coefficient is 0.42, indicating no correlation. Each datapoint in the figure represents a unique biological replicate. All values are presented as the mean ± SD. Statistical analysis was performed with one-way ANOVA. NOR: normoxic; HYP: hypoxic; NC: negative control. *P < 0.05, **P < 0.01, ***P < 0.001 compared with NOR + NC. ^#^p < 0.05, ^##^p < 0.01, ^###^p < 0.001 compared with HYP + NC

It has been reported that many lncRNAs that interact with proteins are essential to a variety of biological processes (Ferre et al. 2016). Therefore, we used AnnoLnc and RNAInter websites to predict proteins associated with FENDRR. Intersection proteins were evaluated by Gene Ontology (GO) and Kyoto Encyclopedia of Genes and Genomes (KEGG) analysis with the DAVID web server, and we found that DNM1L, also known as DRP1, is involved in the NOD-like receptor signaling pathway related to pyroptosis (Fig. 3a). Subsequently, qRT–PCR, Western blot and immunofluorescence assays revealed that upregulation of DRP1 was inhibited by at least 1.5-fold in FENDRR-overexpression HPAECs under hypoxic conditions (Fig. 3b–d). As illustrated by correlation analysis (R^2^ = 0.5439, P < 0.01), FENDRR expression was negatively correlated with DRP1 expression (Fig. 3e). Moreover, to validate whether FENDRR bound to DRP1 in HPAECs, we performed FISH experiments. The statistical significance of Pearson’s coefficient and the result was 0.42 (Pearson’s coefficient > 0.5 was considered meaningful), suggesting that FENDRR did not colocalize well with DRP1 (Fig. 3f). Therefore, we speculated that FENDRR affects cellular functions in another manner.

### FENDRR forms triplexes with the promoter of DRP1 and increases the methylation status of the DRP1 promoter

**Fig. 4:**
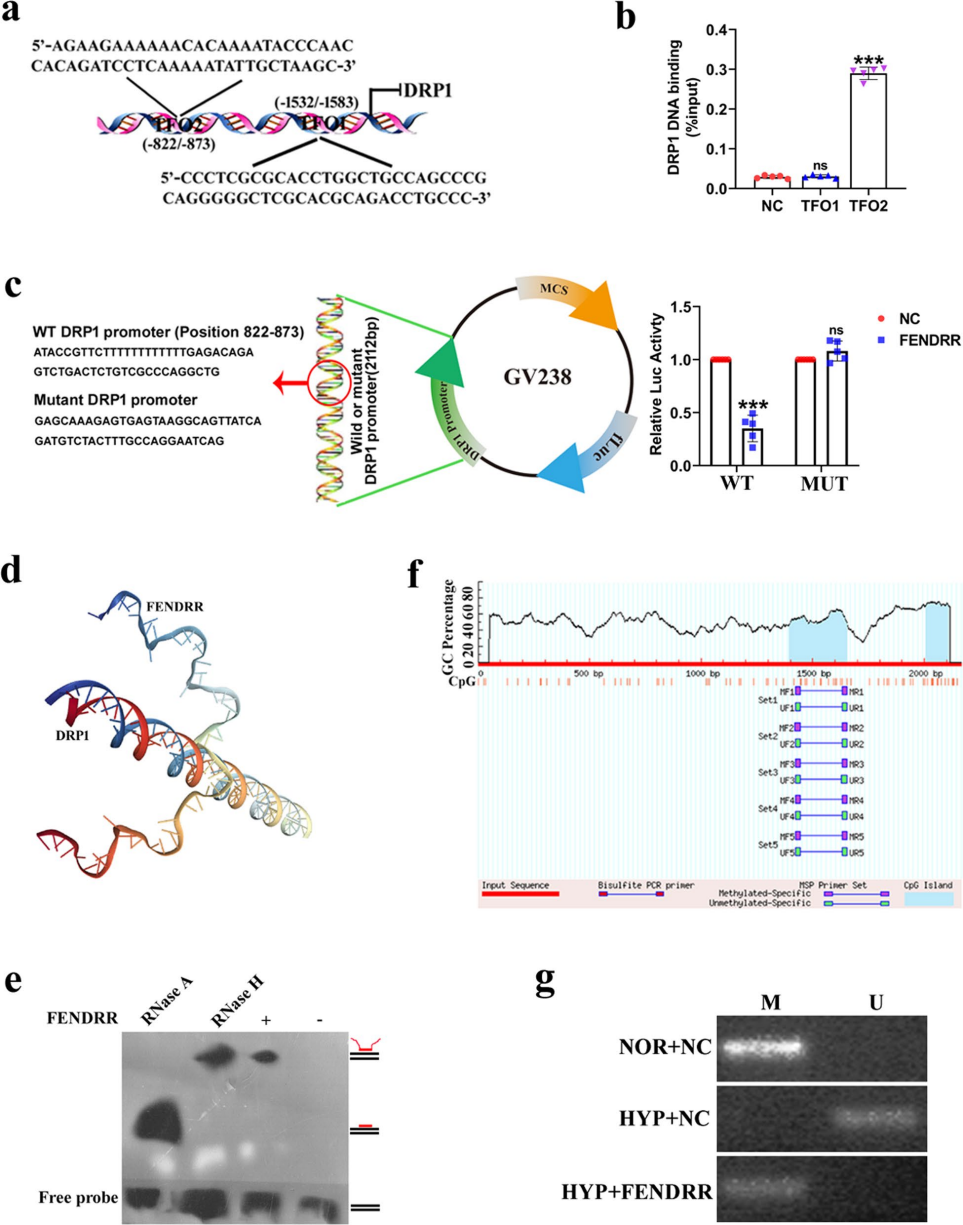
FENDRR affects DRP1 DNA methylation by forming RNA–DNA triplex with DRP1 promoter. **a** Longtarget predicted binding sites of FENDRR and DRP1 promoter. **b** Two triplex forming oligonucleotides (TFOs) within the FENDRR probes and negative control probes were used for ChIRP assay. Purified DNA was analyzed by qPCR (n = 5). **c** HPAECs were cotransfected with a luciferase reporter construct carrying wild-type (WT) or mutant (MUT) DRP1 promoter with FENDRR binding site and FENDRR or NC. Luciferase activities were measured via a dual luciferase assay (n = 5). **d** HNADOCK Server predicted the 3D structural docking of FENDRR and DRP1 promoter. **e** RNA–DNA triplex formed by TFO2 of FENDRR and DRP1 promoter detected by EMSA. **f** Schematic of the CpG islands within the DRP1 promoter. **g** DRP1 promoter methylation status at specific sites in HPAECs transfected with FENDRR overexpression plasmid detected by MSP. Each datapoint in the figure represents a unique biological replicate. All values are presented as the mean ± SD. Statistical analysis was performed with Student’s t-test. NOR: normoxic; HYP: hypoxic; NC: negative control; M: methylation; U: unmethylation; ns: no significant. ***P < 0.001 compared with NC

It has been reported that lncRNAs can interact with gene promoters through Hoogsteen base pairing to form RNA–DNA triplexes, thereby regulating the expression of target genes (Li et al. 2016). Since FENDRR was highly enriched in the nucleus, it is possible that FENDRR interacts with the DRP1 promoter to regulate its expression. Longtarget was used to predict triplex forming oligonucleotides (TFOs) within the FENDRR RNA, and two TFOs with high scores were verified (Fig. 4a). To verify the results, we performed chromatin isolation by RNA purification (CHIRP) assays to explore whether the two TFOs have the ability to bind to the DRP1 promoter. The results showed that DRP1 promoter region was significantly more enriched by tenfold high in the biotin-labeled TFO2 group than that in the NC and biotin-labeled TFO1 groups (Fig. 4b). In addition, a luciferase assay was performed using DRP1 promoter plasmid, containing the DRP1 promoter binding region (WT) of TFO2 and a mutated region (MUT) inserted downstream of a luciferase reporter. The results revealed that the DRP1 promoter WT group obviously decreased luciferase expression compared to the negative control (NC) group approximately threefold after cotransfection with the FENDRR overexpression plasmid, but the DRP1 promoter MUT group showed no notable changes (Fig. 4c). Then, we further used the HNADOCK Server to predict and analyze the 3D structural docking of the FENDRR and DRP1 promoter, and visualized binding complex structure of FENDRR-DRP1 DNA (Fig. 4d). To rule out the possibility of DNA-RNA heteroduplexe formation, RNase H and RNase A were used to analyze the formation of RNA–DNA triplexes by electrophoretic mobility shift assay (EMSA). We found that treatment with RNase H did not affect the mobility of the RNA–DNA complex, so FENDRR could combine with the DRP1 promoter via RNA–DNA triplex formation (Fig. 4e).

As DNA methylation is closely related to the gene transcription process, RNA–DNA triplexes may inhibit target genes through DNA methylation (O'Leary et al. 2015). We suspected that FENDRR might regulate DRP1 transcription in a similar manner. To test this possibility, we found CpG islands in the DRP1 promoter region by using the MethPrimer website (Fig. 4f), suggesting that DNA methylation may exist in DRP1. Accordingly, methylation-specific PCR (MSP) was performed to analyze the methylation level in the promoter region of DRP1 after overexpressing FENDRR. The agarose gel results showed no methylation at the DRP1 promoter region under hypoxia, while methylation was observed in HPAECs overexpressing FENDRR (Fig. 4g). Taken together, these experiments indicated that FENDRR may inhibit DRP1 expression by forming triplexes with the promoter of DRP1 and increasing the methylation status of DRP1.

### DRP1 is involved in FENDRR-mediated HPAEC pyroptosis under hypoxia

**Fig. 5:**
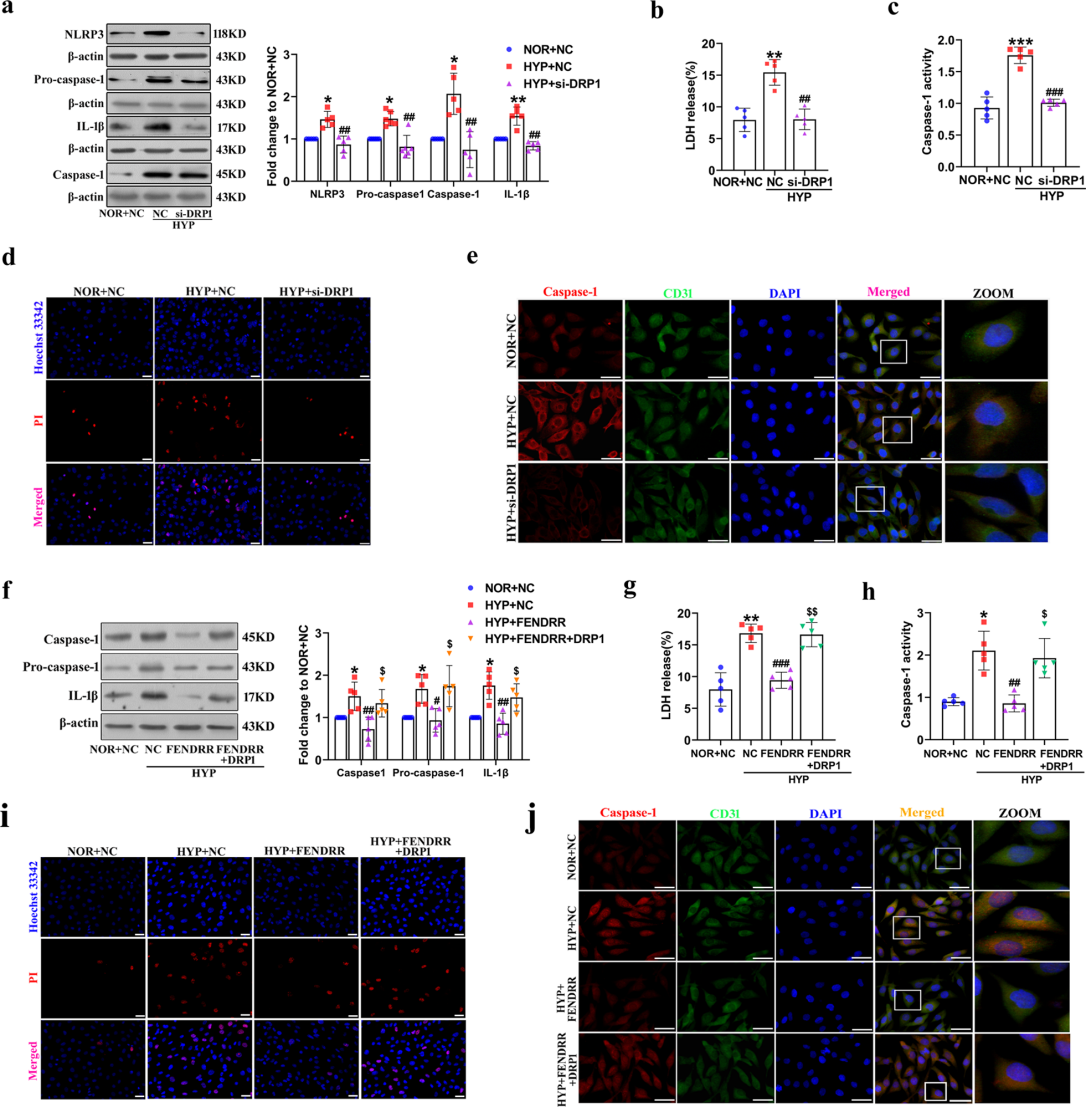
FENDRR regulates HPAECs pyroptosis via DRP1. **a** After transfection with DRP1 siRNA, Western blot was used to examine the protein levels of Caspase-1, NLRP3, Pro-caspase-1 and IL-1β (n = 5). **b** LDH release assays were used to determine the effects of DRP1 on HPAECs pyroptosis (n = 5). **c** The activity of caspase-1 was examined using caspase-1 activity assay kit (n = 5). **d** Images of fluorescence staining with PI (red) and Hoechst 33,342 (blue) were used to detect PI-positive stained cells. Scale bar = 50 μm. **e** Immunofluorescence analysis of Caspase-1 (red) and CD31 (green) expression. Scale bar = 50 μm. **f** After cotransfection with FENDRR and DRP1 overexpression plasmid, Western blotting was used to examine the protein levels of Caspase-1, NLRP3, Pro-caspase-1 and IL-1β (n = 5). **g** and **h** LDH release assays and activity of caspase-1 were used to determine the effects of cotransfection with FENDRR and DRP1 overexpression plasmid on HPAECs pyroptosis (n = 5). **i** and **j** PI staining and fluorescence intensity of Caspase-1 were used to determine the effects of cotransfection with FENDRR and DRP1 overexpression plasmid on HPAECs pyroptosis. Each datapoint in the figure represents a unique biological replicate. All values are presented as the mean ± SD. Statistical analysis was performed with one-way ANOVA. NOR: normoxic; HYP: hypoxic; NC: negative control. **P < 0.01, *P < 0.05 compared with NOR + NC. ^#^p < 0.05, ^##^p < 0.01, ^###^p < 0.001 versus HYP + NC. ^$^p < 0.05, ^$$^p < 0.01 compared with HYP + FENDRR

Previous studies have shown that upregulation of DRP1 is involved in pulmonary vascular remodeling in HPH by controlling metabolic pathways and the proliferation of PASMCs and PAECs (Ryan et al. 2015; Chen et al. 2018; Shen et al. 2015). However, whether DRP1 regulates hypoxia-induced HPAEC pyroptosis has not been reported. We transfected DRP1 siRNA into HPAECs to knock down DRP1 protein expression, interference efficiency was 70% (Additional file 1: Fig. S4a). The increased expression of the proteins NLRP3, Caspase-1, pro-Caspase-1 and IL-1β under hypoxia was reduced by DRP1 siRNA (Fig. 5a). We further observed that DRP1 siRNA decreased the level of LDH activity from 15.4% to 8.0% under hypoxic conditions (Fig. 5b). Caspase-1 activity levels also were decreased by DRP1 siRNA in hypoxia HPAECs, the relative values are 0.93 ± 0.17, 1.76 ± 0.13, 1.01 ± 0.06 (Fig. 5c). Moreover, PI staining and fluorescence intensity of Caspase-1 was observed to be increased under hypoxia exposure and reversed by silencing the DRP1 gene (Fig. 5d and e).

Finally, we performed rescue experiments on cell pyroptosis to determine whether DRP1 is involved in FENDRR-mediated HPAEC pyroptosis. DRP1 protein expression was significantly upregulated by fourfold high after transfection of the DRP1 overexpression plasmid into HPAECs (Additional file 1: Fig. S4b). Overexpression of DRP1 partially restored the increased levels of pyroptosis-related proteins inhibited by FENDRR overexpression under hypoxia (Fig. 5f). LDH release assay demonstrated that the FENDRR overexpression-induced decrease LDH release was rescued by DRP1 overexpression under hypoxic conditions, LDH release value are 7.98%, 16.83%, 9.43%, 16.61% (Fig. 5g). In addition, this FENDRR-mediated attenuation of hypoxia-induced PAEC Caspase-1 activity levels could be rescued by DRP1 overexpression, Caspase-1 activity relative values are 0.90 ± 0.09, 2.11 ± 0.46, 0.86 ± 0.19, 1.93 ± 0.46 (Fig. 5h). Similar results were observed in PI staining and immunofluorescence of Caspase-1 assays (Fig. 5i and j). These data indicated that DRP1, as a downstream target gene of FENDRR, is involved in FENDRR-mediated HPAEC pyroptosis under hypoxia.

### YTHDC1 bound m6A modified FENDRR and decreased its stability

**Fig. 6:**
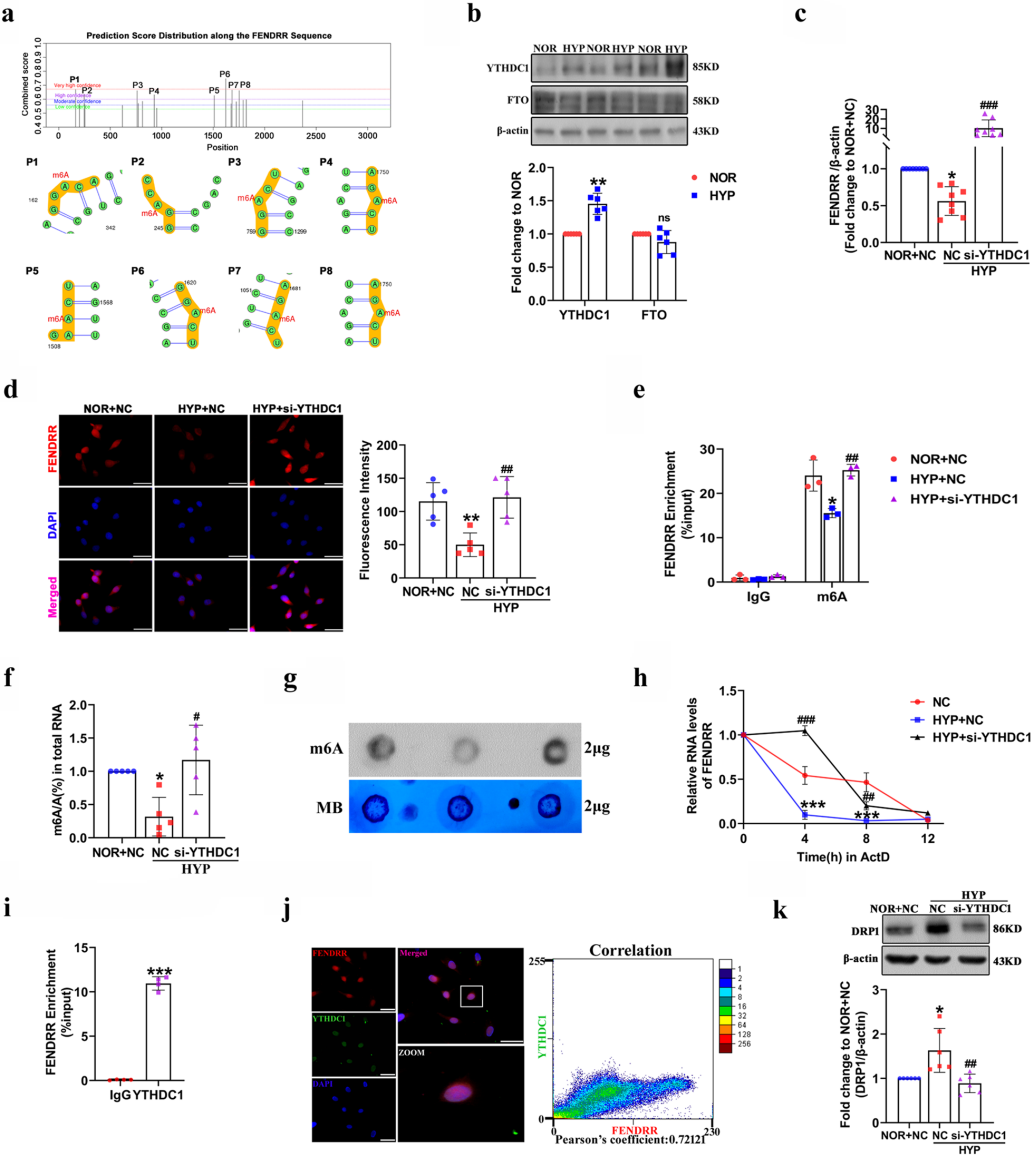
YTHDC1 binds m6A modified FENDRR to decrease its stability. **a** SRAMP predicted m6A sites of FENDRR. **b** Western blotting was used to verify the expression of YTHDC1 and FTO under hypoxic conditions (n = 6). **c** qRT-PCR detection of FENDRR expression (n = 8). **d** FISH assay was performed to detect FENDRR expression. Scale bars = 50 µm. FENDRR probes were labeled with Cy3 (red) and nuclei were stained with DAPI (blue). **e** The m6A levels of FENDRR were quantified by MeRIP followed by qRT–PCR (n = 3). **f** and **g** EpiQuik m6A RNA Methylation Quantification Kit and Dot blot were used to detect global m6A modifications in total HPAECs RNAs (n = 5). **h** The qRT-PCR analysis of FENDRR levels after ActD (1 µg/mL) treatment at 0 h, 4 h, 8 h and 12 h (n = 4). **i** RNA RIP assay was performed to detect FENDRR interacts with the YTHDC1 protein (n = 4). **j** Colocalization of FENDRR and YTHDC1 in HPAECs. Scale bars = 50 µm. FENDRR probes were labeled with Cy3 (red), YTHDC1 were stained with FITC (green) and nuclei were stained with DAPI (blue). Pearson coefficient is 0.72121, indicating correlation. **k** After transfection with YTHDC1 siRNA, Western blotting was used to examine the protein levels of DRP1 (n = 6). Each datapoint in the figure represents a unique biological replicate. All values are presented as the mean ± SD. Statistical analysis was performed with one-way ANOVA or the Student’s t-test. NOR: normoxic; HYP: hypoxic; NC: negative control; ns: no significant. *P < 0.05, **P < 0.01, ***P < 0.001 compared with NOR + NC. ^###^p < 0.001, ^##^p < 0.01, ^#^p < 0.05 compared with HYP + NC

Recent studies have suggested that m6A RNA modification is a novel mediator of pathological changes in PH by regulating diverse transcripts (Hu et al. 2021; Xu et al. 2021). Therefore, we attempted to explore how m6A affects the FENDRR transcript under hypoxic conditions. To address this issue, we verified the m6A modification of FENDRR by using the SRAMP prediction server, and found eight high confidence m6A modification sites of FENDRR (Fig. 6a). The FENDRR target proteins predicted in Fig. 4a contained several m6A modification enzymes, including FTO and YTHDC1 located in the nucleus. Next, YTHDC1 and FTO expression in HPAECs was evaluated using Western blot. The results showed that the expression of YTHDC1 was enriched by 1.4-fold high in HPAECs under hypoxic conditions; FTO did not change significantly. Therefore, we chose YTHDC1 for subsequent analyses (Fig. 6b). We transfected YTHDC1 siRNA into HPAECs to silence YTHDC1, and si-1 had the most obvious effects, interference efficiency was 50% (Additional file 1: Fig. S5a and b). To clarify whether YTHDC1 affects FENDRR expression under hypoxia exposure, HPAECs were transfected with YTHDC1 siRNA. qRT–PCR showed decreased expression of FENDRR under hypoxia, which was reversed by YTHDC1 siRNA transfection, the relative values are 1.00 ± 0.00, 0.56 ± 0.19, 10.2 ± 8.88 (Fig. 6c). Similar results were observed in the FISH experiment, suggesting that the downregulation of FENDRR was mediated by YTHDC1 in hypoxia (Fig. 6d). In addition, MeRIP-PCR results showed that the m6A level of FENDRR was increased by 1.6-fold high in YTHDC1-silenced HPAECs compared with that in the NC group under hypoxia (Fig. 6e). We further detected global m6A RNA modification in HPAECs using the EpiQuik™ m6A RNA Methylation Quantification Kit (Colorimetric, P-9005-48, USA), the results showed that the global m6A RNA modification was increased by 3.7-fold high in YTHDC1-silenced HPAECs compared with that in the NC group under hypoxia (Fig. 6f). Similar trends in dot blot were found in HPAECs after YTHDC1 siRNA treatment (Fig. 6g). Studies have pointed out that YTHDC1 can regulate RNA stability (Liang 2021), so the half-life of FENDRR was tested by incubation with the transcription inhibitor actinomycin D on YTHDC1-silenced HPAECs, and RNA was obtained at different time points. Indeed, silencing YTHDC1 significantly prolonged the lifetime of FENDRR under hypoxic conditions (Fig. 6h). Therefore, YTHDC1 decreased FENDRR stability, indicating the leading role of YTHDC1 in the m6A-mediated degradation process of FENDRR.

To test the interaction between FENDRR and YTHDC1, we performed a RIP assay, and the result suggested that YTHDC1 was significantly enriched by tenfold in FENDRR RNA to form an m6A modification complex (Fig. 6i). Fluorescence colocalization assays revealed that FFNEDRR and YTHDC1 were colocalized in the nuclei of HPAEC, the statistical significance of Pearson’s coefficient and the result was 0.72 (Pearson’s coefficient > 0.5 was considered meaningful) (Fig. 6j). Moreover, Western blot indicated that YTHDC1 siRNA reduced the level of DRP1 compared to hypoxia, the relative values are 1.00 ± 0.00, 1.63 ± 0.49, 0.89 ± 0.21 (Fig. 6k). In conclusion, these results showed that YTHDC1 selectively binds to m6A-modified FENDRR and modulates its degradation in an m6A-dependent manner, providing a novel view for the dysregulation of FENDRR in HPH progression.

### The conserved TFO2 sequence of FENDRR participates in HPH through pyroptosis in hypoxic mouse model

**Fig. 7:**
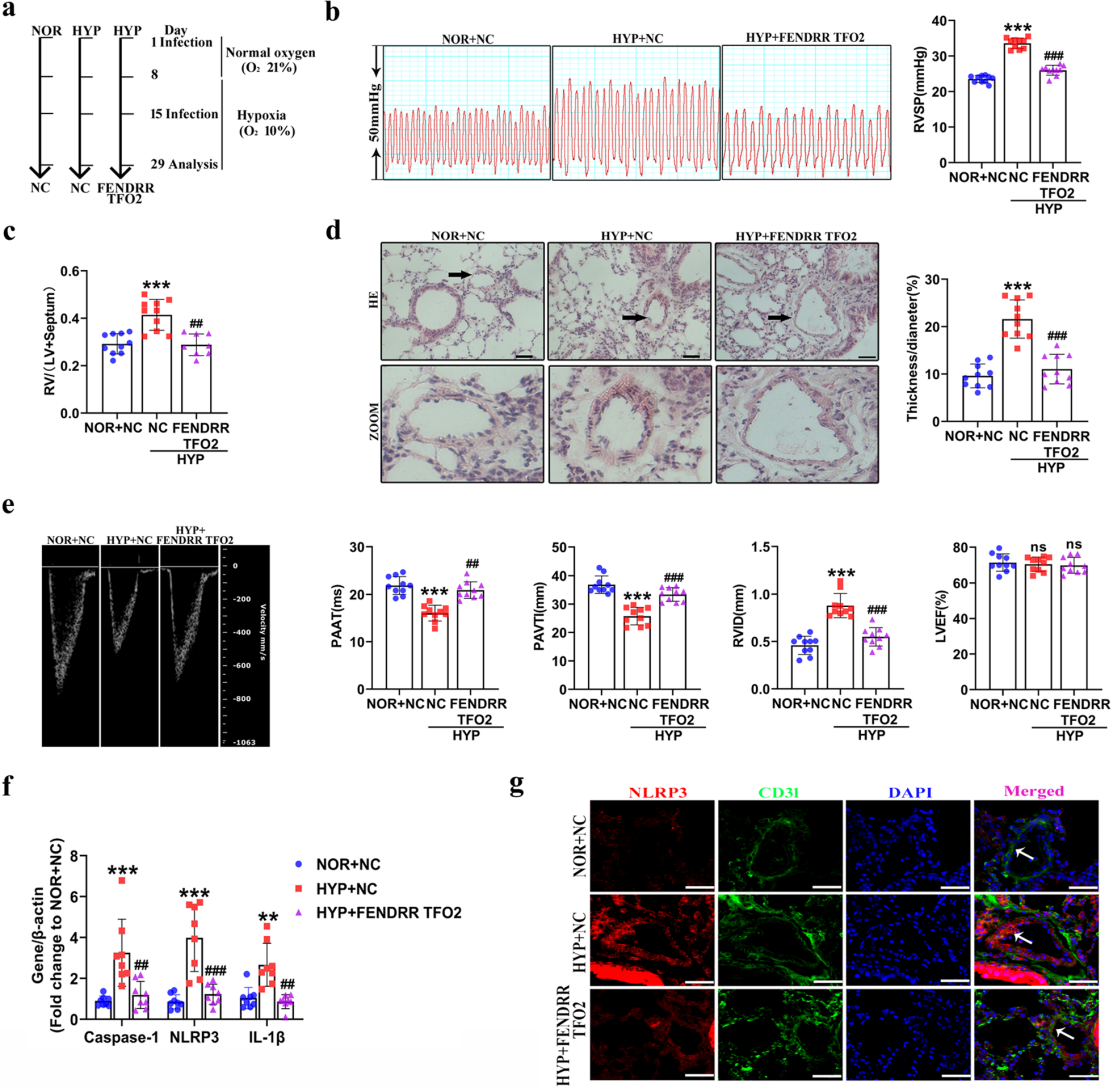
Overexpression conserved sequence TFO2 adenovirus of FENDRR in vivo alleviates the development of HPH and inhibits hypoxia-induced pyroptosis. **a** Adenoviruses with NC or TFO2 sequences were used to handle mice via dropwise intranasal instillation to mice and subjected to hypoxia until 29 days. **b** Indices of Right ventricular systolic pressure (RVSP) (n = 10). **c** Right ventricle (RV)/(left ventricle (LV) + Septum) weight ratio was calculated (n = 10). **d** HE staining was performed to detect wall thickening (n = 10). **e** Echocardiographic images and indexes (n = 10). **f** qRT–PCR was used to examine the lung tissues mRNA levels of Caspase-1, NLRP3 and IL-1β (n = 8). **g** Immunofluorescence of NLRP3 in mouse lung sections. Scale bars = 50 µm. All values are presented as the mean ± SD. Statistical analysis was performed with one-way ANOVA. NOR: normoxic; HYP: hypoxic; NC: negative control; ns: no significant. *P < 0.05, **P < 0.01, ***P < 0.001 compared with NOR + NC. ^#^p < 0.05, ^##^p < 0.01, ^###^p < 0.001 compared with HYP + NC

Although we did not find the mouse homolog of FENDRR, the functional fragment TFO2 of FENDRR (464–516) showed higher conservation of the sequence among humans and mice (Additional file 1: Fig. S6a). To evaluate the function of the conserved TFO2 sequences of FENDRR in the HPH mouse model, adenoviruses with NC or TFO2 sequences were used to treat mice via dropwise intranasal instillation (Fig. 7a). Subsequently, TFO2 overexpression was verified by in situ hybridization methods (Additional file 1: Fig. S6b). Next, we explored whether TFO2 overexpression prevented hypoxia-induced PH in vivo. We characterized the mice in detail, including right ventricular systolic pressure (RVSP), RV/left ventricular (LV) + Septum weight ratio, hemodynamics, cardiac function, and vascular remodeling. The results illustrated that overexpression of the TFO2 fragment inhibited hypoxia-induced RVSP and RV/(LV + S) (Fig. 7b and c). Moreover, we found that TFO2 overexpression reversed hypoxia-induced pulmonary vascular remodeling by HE staining (Fig. 7d). Echocardiographic analysis showed that the pulmonary artery acceleration time (PAAT) and pulmonary arterial velocity time integral (PAVTI) were significantly decreased, and the right ventricle internal diameter (RVID) was increased under hypoxia in mice, while the effect was prevented by TFO2 overexpression. Importantly, the left ventricular ejection fraction (LVEF) was not changed in mice with or without TFO2 overexpression (Fig. 7e).

In addition, we examined the effect of TFO2 overexpression on pyroptosis in lung tissues, and mRNA expression levels of pyroptosis-related genes were significantly lower in TFO2 overexpressing mouse lung tissues than in NC-treated mouse lung tissues after hypoxia exposure (Fig. 7f). Immunofluorescence staining for NLRP3 revealed that the fluorescence activity in TFO2 overexpressing mouse lung tissues was decreased compared with that in NC-treated mouse lung tissues after hypoxia exposure (Fig. 7g). Similarly, DRP1-positive staining was inhibited by TFO2 overexpressing under hypoxic conditions (Additional file 1: Fig. S6c). Western blot also indicated that TFO2 overexpressing reduced the protein level of DRP1 compared to hypoxia, the relative values are 0.77 ± 0.24, 1.60 ± 0.11, 0.88 ± 0.32 (Additional file 1: Fig. S6d). To verify the effect of TFO2 adenovirus on PH under normoxic conditions, adenoviruses with NC or TFO2 sequences were used to treat mice via dropwise intranasal instillation (Additional file 1: Fig. S7a). Subsequently, TFO2 overexpression was verified by in situ hybridization methods, TFO2-positive staining was twofold higher than the NOR + NC group (Additional file 1: Fig. S7b). Both the RVSP, RV/left ventricular (LV) + Septum weight ratio and vascular remodeling were unchanged in NOR + NC group and NOR + FENDRR TFO2 group (Additional file 1: Fig. S7c–e). Consistent with these results, echocardiographic analysis showed that the PAAT, PAVTA and LVEF were not changed in mice with or without TFO2 overexpression at baseline conditions (Additional file 1: Fig. S7f). These results confirmed that the conserved TFO2 sequence of FENDRR might regulate the pathology of HPH through pyroptosis in vivo.

## Discussion

In the current study, we proved that FENDRR was localized in the nucleus of PAECs and downregulated in response to hypoxia. Our results support that FENDRR plays a key role in hypoxia-induced HPAECs pyroptosis via regulation of the downstream target DRP1. Mechanistically, FENDRR formed an RNA–DNA triplex within the promoter of DRP1, leading to decreased transcription of DRP1 by promoting DRP1 promoter methylation. More importantly, we showed a new mechanism of FENDRR degradation through binding to the m6A “reader” YTHDC1. In addition, the fragment of the TFO2 sequence (464–516) of FENDRR was the pivotal functional domain that interacted with the DRP1 promoter. The conserved fragment of TFO2 of FENDRR might reverse pyroptosis in an HPH mouse model. These findings implicate FENDRR in PAEC pyroptosis induced by hypoxia, which is a novel mediator in HPH.

Increasing evidence suggests that pyroptosis is implicated in cardiovascular diseases, including HPH. It has been reported that PASMC pyroptosis is mediated by circCalm4 and Krüppel zinc finger protein GLI1 in HPH (Jiang et al. 2021b; He et al. 2020b). Moreover, the activation of pyroptosis in PASMCs, which is related to pulmonary fibrosis induced by hypoxia, was alleviated by treatment with Caspase-1 inhibitors (Zhang et al. 2020). However, whether pyroptosis of PAECs is involved in HPH remains largely unknown. In this study, our findings demonstrated that the pyroptosis-related markers NLRP3, Caspase-1 pro-caspase-1 and IL-1β were highly upregulated in hypoxic PAECs. In addition, PI staining and LDH activity increased in PAECs under hypoxic conditions. Therefore, our results provide solid evidence that pyroptosis can occur in PAECs treated with hypoxia, and play a potential role in the development of HPH.

Understandably, one of the mechanisms of lncRNAs in the regulation of their biological functions occurs through binding to DNA or proteins in the nucleus to regulate gene transcription and splicing levels (Sun et al. 2018). In the present study, we used bioinformatics analysis to predict proteins with potential sites for binding to FENDRR, and overexpression of FENDRR decreased the mRNA and protein level of DRP1 upon hypoxia exposure, suggesting that DRP1 expression is regulated by FENDRR in PAECs. The possibility of FENDRR and DRP1 protein interaction was ruled out, since the binding tendency between them, as shown by fluorescence colocalization, was very low. We further identified that FENDRR can interact with the DRP1 promoter by performing CHIRP assays and EMSAs. In addition, we confirmed that FENDRR mediated PAEC pyroptosis via DRP1. Therefore, we provide an effective mechanism by which nuclear FENDRR can form RNA–DNA triplexes with the DRP1 promoter, and downregulate the expression of DRP1, thereby inhibiting the occurrence of PAEC pyroptosis. Interestingly, some studies have demonstrated that FENDRR in the cytoplasm participates in the progression of cancer through the ceRNA mechanism (Yu et al. 2019; Cheng et al. 2020), suggesting that FENDRR may play a different role according to its subcellular compartment environment.

Recent studies have demonstrated that RNA–DNA triplexes regulate target gene transcription activity by influencing CpG island methylation or transcription factors (Li et al. 2019). In our study, we found that FENDRR forms an RNA–DNA triplex with the DRP1 promoter to enhance the methylation status of the DRP1 promoter CpG island, which inhibits the transcription of DRP1. From the perspective of nuclear FENDRR regulation, we verified that the RNA–DNA triplex structure affects gene transcription through DNA methylation as an epigenetic mechanism underlying the regulatory role of FENDRR in PAEC pyroptosis induced by hypoxia.

DPR1 was originally known as a key fission protein in mitochondrial dynamics, and is involved in pathological proliferation and apoptosis resistance of pulmonary vasculature in HPH (Chen et al. 2019; Zhang et al. 2016). Some studies have shown that DRP1 can regulate the occurrence of pyroptosis by influencing mitochondrial homeostasis (Zou et al. 2020). Our present study revealed that DRP1, which regulates hypoxia-induced PAEC pyroptosis, was reversed by FENDRR overexpression in hypoxia-exposed PAECs. More importantly, we observed that overexpression of FENDRR cannot inhibit the generation of mitochondrial reactive oxygen species (ROS) in PAECs caused by hypoxia (Additional file 1: Fig. S8a). Therefore, FENDRR decreased hypoxia-induced PAEC pyroptosis via DRP1 without relying on the regulation of mitochondrial function, suggesting that DRP1 not only has important functions in the mitochondria but also plays a regulatory role as a cytokine in the cytoplasm.

N6-methyladenosine (m6A) is the most abundant chemical RNA modification in mRNA and noncoding RNA, and regulates multiple biological processes, such as cell proliferation, migration and tumorigenesis (Liu et al. 2020b). Emerging studies have shown that m6A methylation modification can occur in HPH (Xu et al. 2021). However, there are no studies on the function of m6A in lncRNAs associated with HPH. In this study, we revealed that the m6A “reader” YTHDC1 might negatively regulate FENDRR stability by directly combining with FENDRR. YTHDC1 siRNA significantly prolonged the decay rate of FENDRR as a result of the accumulation of m6A modifications in FENDRR in hypoxic PAECs. These results suggested that YTHDC1 targeted the m6A sites of FENDRR and subsequently decreased FENDRR expression. This is a novel finding regarding the upstream regulatory mechanism of FENDRR under hypoxic conditions.

## Conclusions

**Fig. 8:**
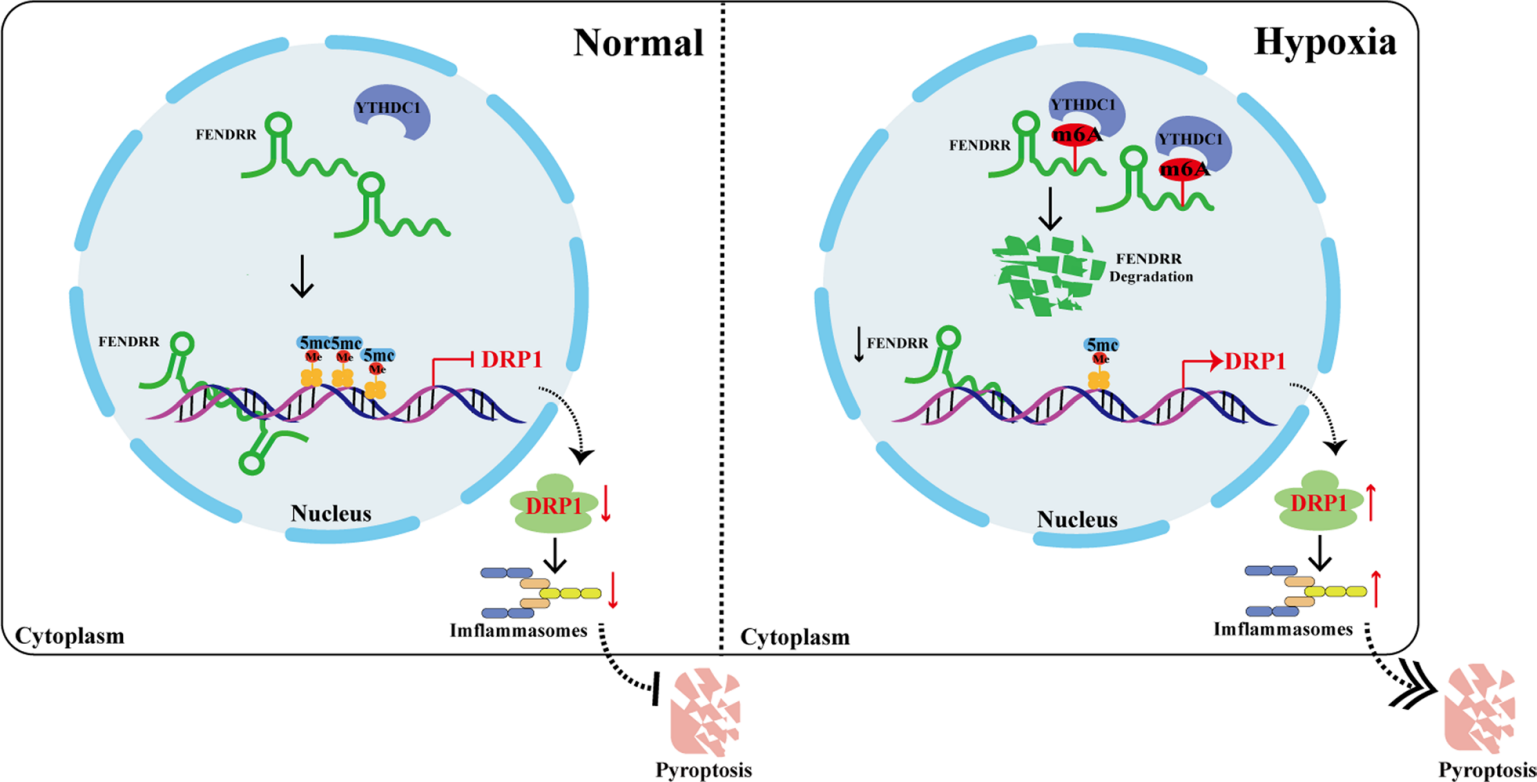
A schematic diagram to illustrate the hypothetical model

To conclude, the present study uncovered the involvement of m6A “reader” YTHDC1-medited nuclear FENDRR in hypoxia-induced PAEC pyroptosis by forming RNA–DNA triplex with DRP1 promoter to promote its methylation at CpG islands, along with changes in transcriptional activity, inhibiting the expression of DRP1 (Fig. 8). Our results provide new clues for the study of the molecular regulatory mechanism of pyroptosis in PAECs and may provide potential therapeutic targets in HPH.

## Supplementary Information

**Additional file 1:** **Fig. S1. **Specific primer of FENDRR were designed by NCBI; subcellular localization of FENDRR predicted by lncATLAS website and secondary structure of FENDRR. **Fig. S2.** Overexpression efficiency of FENDRR; the protein levels of Caspase-4 and Caspase-11. **Fig. S3.** FENDRR ASO enhances cell pyroptosis in HPAECs. **Fig. S4.** Interference efficiency and overexpression efficiency of DRP1. **Fig. S5.** Interference efficiency of YTHDC1. **Fig. S6.** Conservative analysis of the functional fragment TFO2 of FENDRR (464–516); in situ hybridization of the functional fragment TFO2 of FENDRR (464–516); the expression levels of DRP1 were detected by immunofluorescence and western blotting. **Fig. S7.** Overexpression conserved sequence TFO2 adenovirus of FENDRR in vivo does not affect the development of PH under normoxic conditions. **Fig. S8.** Mitochondrial superoxide indicator (Mito-SOX Red) was used to detect the mitochondrial-derived ROS production.

## Data Availability

The datasets used and/or analysed during the current study are available from the corresponding author on reasonable request.

## Abbreviations

HPH: Hypoxic pulmonary hypertension
HPAECs: Human pulmonary artery endothelial cells
HPASMCs: Human pulmonary artery smooth muscle cells
IGF1R: Type 1 insulin-like growth factor receptor
TIMP2: Tissue inhibitor of metalloproteinase 2
m6A: N6-methyladenosine
DRP1: Dynamin-related protein 1
ASOs: Antisense oligonucleotides
TFOs: Triplex forming oligonucleotides
CHIRP: Chromatin isolation by RNA purification
EMSA: Electrophoretic mobility shift assay
MSP: Methylation-specific PCR
ROS: Reactive oxygen species
