# Supplementary material for: LncRNA FENDRR with m6A RNA methylation regulates hypoxia-induced pulmonary artery endothelial cell pyroptosis by mediating DRP1 DNA methylation
Source: Mol Med. 2022 Oct 25;28:126. doi: 10.1186/s10020-022-00551-z (PMC9594874; doi:10.1186/s10020-022-00551-z)
Supplement: Supplementary file 1 — Additional file 1: Fig. S1. Specific primer of FENDRR were designed by NCBI; subcellular localization of FENDRR predicted by lncATLAS website and secondary structure of FENDRR. Fig. S2. Overexpression efficiency of FENDRR; the protein levels of Caspase-4 and Caspase-11. Fig. S3. FENDRR ASO enhances cell pyroptosis in HPAECs. Fig. S4. Interference efficiency and overexpression efficiency of DRP1. Fig. S5. Interference efficiency of YTHDC1. Fig. S6. Conservative analysis of the functional fragment TFO2 of FENDRR (464–516); in situ hybridization of the functional fragment TFO2 of FENDRR (464–516); the expression levels of DRP1 were detected by immunofluorescence and western blotting. Fig. S7. Overexpression conserved sequence TFO2 adenovirus of FENDRR in vivo does not affect the development of PH under normoxic conditions. Fig. S8. Mitochondrial superoxide indicator (Mito-SOX Red) was used to detect the mitochondrial-derived ROS production. [file 10020_2022_551_MOESM1_ESM.docx]

**LncRNA FENDRR with m6A RNA methylation regulates hypoxia-induced pulmonary artery endothelial cell pyroptosis by mediating DRP1 DNA methylation**

Xiaoying Wang^1,2#^·Qian Li^2#^·Siyu He^1,2^·June Bai^1,2^·Cui Ma^1,4^·Lixin Zhang^1,4^·Xiaoyu Guan^1,2^·Hao Yuan^1,2^·Yiying Li^1,2^·Xiangrui Zhu^4^·Jian Mei^4^·Feng Gao^5^·Daling Zhu^1,2,3*^

^1^Central Laboratory of Harbin Medical University (Daqing), Daqing 163319, P. R. China

^2^College of Pharmacy, Harbin Medical University, Harbin, 150081, P. R. China

^3^Key Laboratory of Cardiovascular Medicine Research, Ministry of Education, Harbin Medical University, Harbin, 150081, P. R. China

^4^College of Medical Laboratory Science and Technology, Harbin Medical University (Daqing), Daqing 163319, P. R. China

^5^Midwestern University, College of Dental Medicine-Illinois, Downers Grove, IL 60515, USA

**^#^** These authors contributed equally to this work.

^*^Corresponding Author: Dr. Daling Zhu, College of Pharmacy, Harbin Medical University (Daqing), Xinyang Road Daqing, Heilongjiang 163319, P. R. China, Tel: 011-86-459-8153555, Fax: 011-86-459-8153556, E-mail: zhudaling@hrbmu.edu.cn.

**
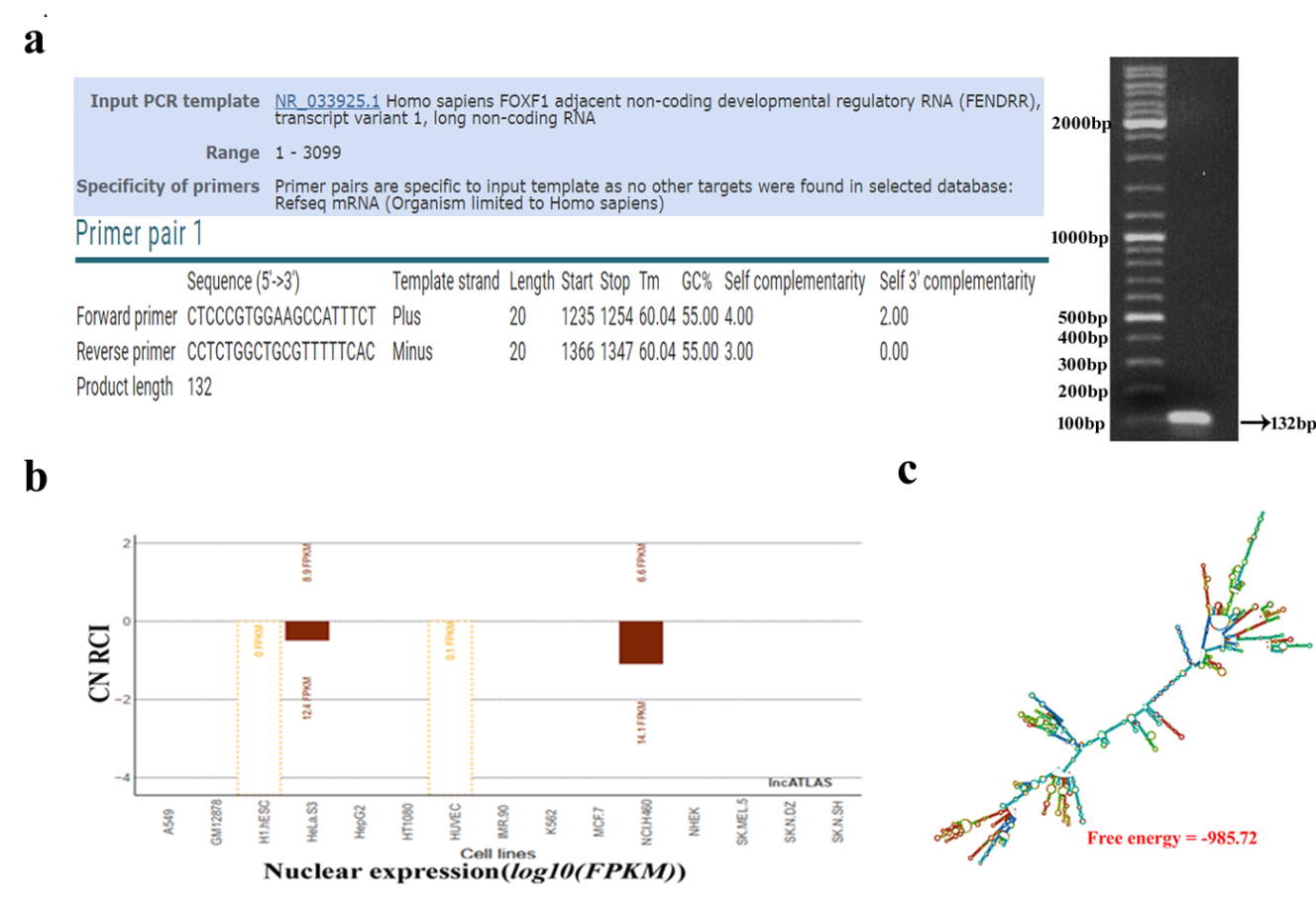
**

**Additional Fig. S1**. **a** Specific primer of FENDRR were designed by NCBI. The product length was confirmed to be 132bp by agarose gel electrophoresis assay. **b** Subcellular localization of FENDRR predicted by lncATLAS website. **c** The RNAfold Web server was used to predict the secondary structure of FENDRR.


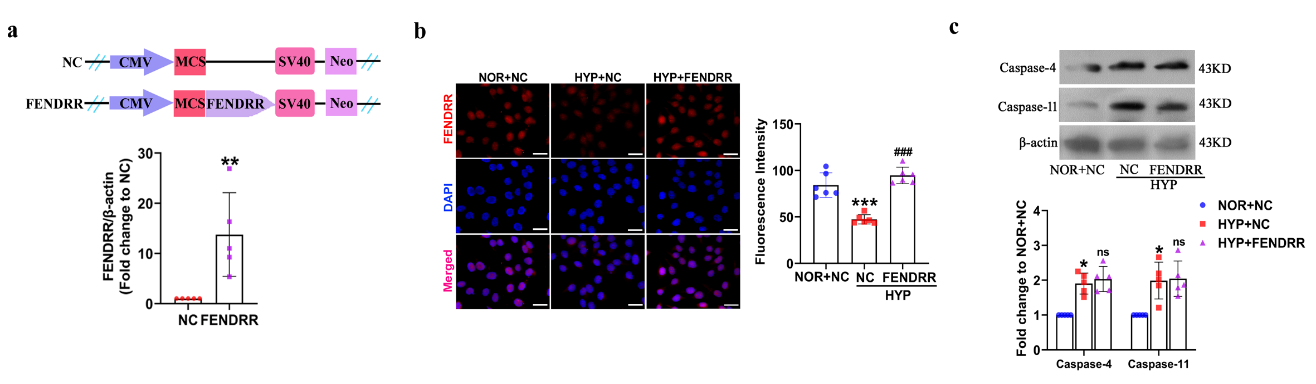


**Additional Fig. S2** **a** Overexpression efficiency of FENDRR were quantified by qRT–PCR (n=5). **b** FISH assay verified that FENDRR is overexpressed in the nucleus. FENDRR probes were labeled with Cy3 (red) and nuclei were stained with DAPI (blue). **c** Overexpression FENDRR did not affect the increased protein levels of Caspase-4 and Caspase-11 induced by hypoxia in HPAECs (n = 5). All values are presented as the mean ± SD. Each datapoint in the figure represents a unique biological replicate. Statistical analysis was performed with the Student’s t-test. NOR: normoxic; HYP: hypoxic; NC: negative control; ns: no significant. *P < 0.05, **P < 0.01, ***P < 0.001 compared with NOR + NC, ^###^p < 0.001 compared with HYP + NC.


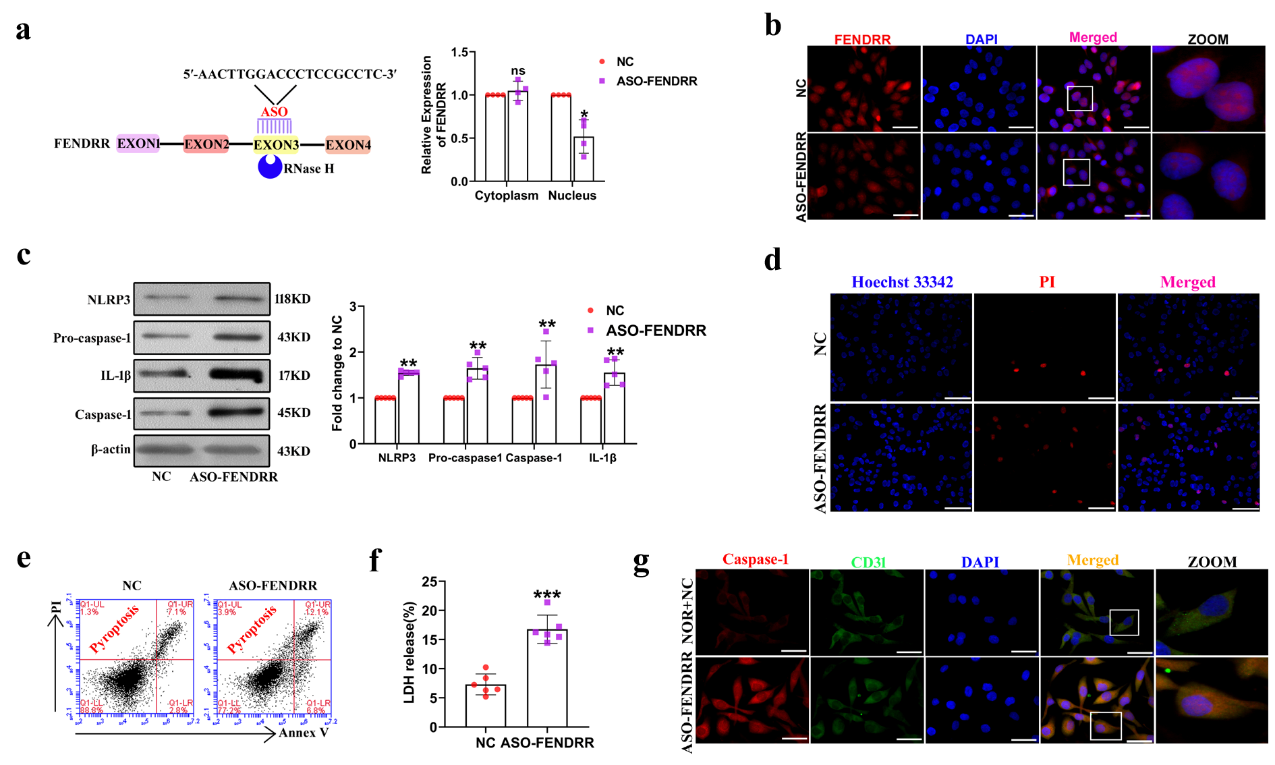


**Additional Fig. S3** FENDRR ASO enhances cell pyroptosis in HPAECs. **a** Interference efficiency of FENDRR ASO were quantified by qRT–PCR (n = 4). **b** FISH experiment verified that FENDRR ASO interfere with FENDEE in the nucleus. FENDRR probes were labeled with Cy3 (red) and nuclei were stained with DAPI (blue) Scale bar = 50 μm. **c** After transfection with FENDRR ASO, Western blotting was used to examine the protein levels of Caspase-1, NLRP3, Pro-caspase-1 and IL-1β (n = 5). **d** Images of fluorescence staining with PI (red) and Hoechst 33342 (blue) were used to detect PI-positive stained cells. Scale bar = 50 μm. **e** The Flow cytometry assay was used to detect the population of positive-PI cells. HPAECs were treated with annexin V-FITC/propidium iodide (PI) double staining using quantitative fluorescence-activated cell sorting (FACS) analysis. **f** LDH release assays were used to determine the effects of FENDRR ASO on HPAECs pyroptosis (n = 6). **g** Immunofluorescence analysis of Caspase-1 (red) and CD31 (green) expression. Scale bar = 50 μm. All values are presented as the mean ± SD. Each datapoint in the figure represents a unique biological replicate. Statistical analysis was performed with the Student’s t-test. NC: negative control; ns: no significant. *P < 0.05, **P < 0.01, ***P < 0.001 compared with NC.


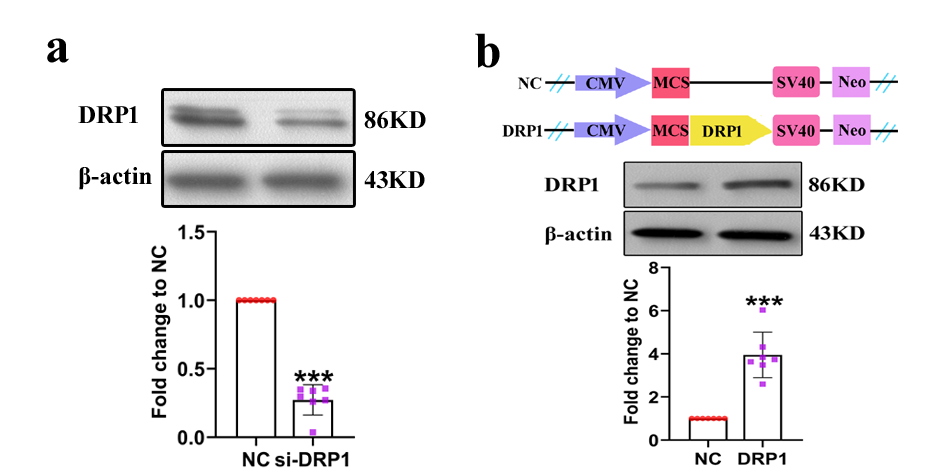


**Additional Fig. S4** **a** Interference efficiency of DRP1 were quantified by Western blotting (n = 6). **b** Overexpression efficiency of DRP1 were quantified by Western blotting (n = 7). Each datapoint in the figure represents a unique biological replicate. All values are presented as the mean ± SD. Statistical analysis was performed with the Student’s t-test. ***P < 0.001 compared with NC.


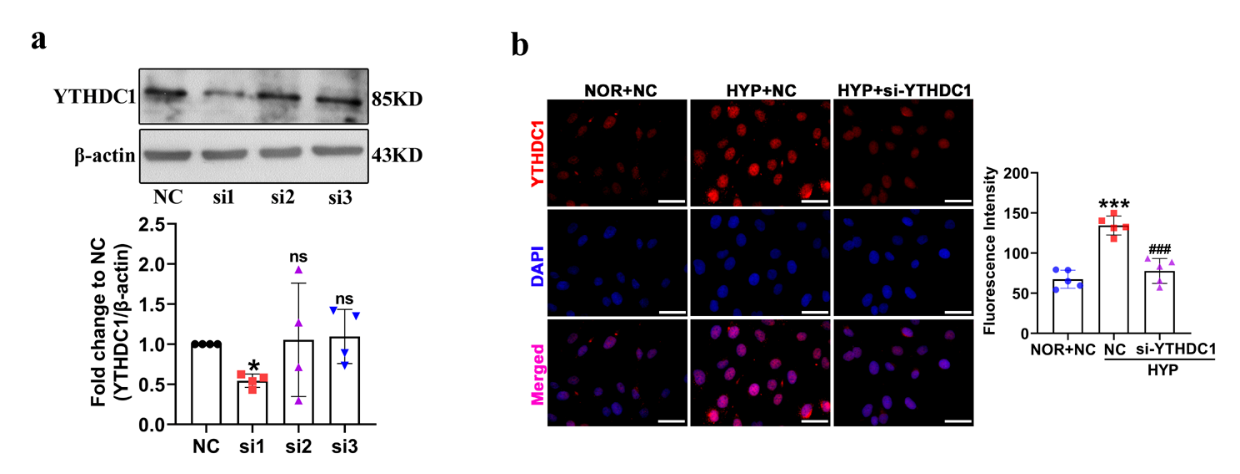


**Additional Fig. S5** **a** Interference efficiency of YTHDC1 were quantified by Western blotting (n = 4). **b** Fluorescence staining for YTHDC1. HPAECs were stained for YTHDC1 (red) and DAPI (blue) was used for nuclear staining. Scale bar = 50 μm. Each datapoint in the figure represents a unique biological replicate. All values are presented as the mean ± SD. Statistical analysis was performed with the Student’s t-test. NC: negative control; ns: no significant. *P < 0.05 compared with NC.


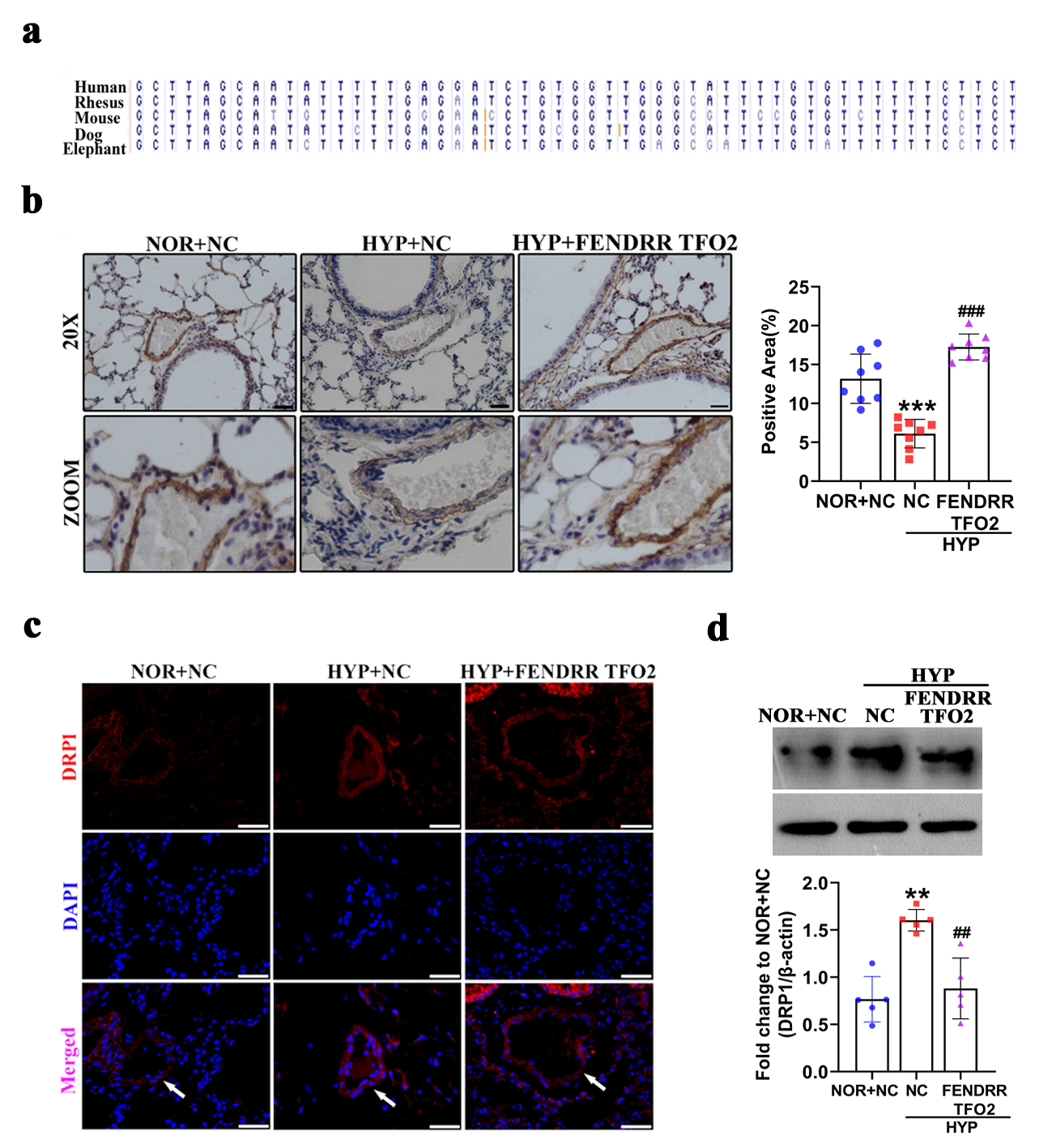


**Additional Fig. S6** **a** Conservative analysis of the functional fragment TFO2 of FENDRR (464–516) by UCSC Genome Browser. **b** The functional fragment TFO2 of FENDRR overexpression was verified by in situ hybridization methods (n = 8). Scale bar = 100 μm. **c** Immunofluorescence of DRP1 in mouse lung sections. Scale bars=50 µm. **d** Western blotting was used to examine the protein levels of DRP1 in mouse lung tissues (n = 5). All values are presented as the mean ± SD. Statistical analysis was performed with one-way ANOVA. NOR: normoxic; HYP: hypoxic; NC: negative control. **P < 0.01 compared with NOR + NC. ^##^p < 0.01 compared with HYP + NC.


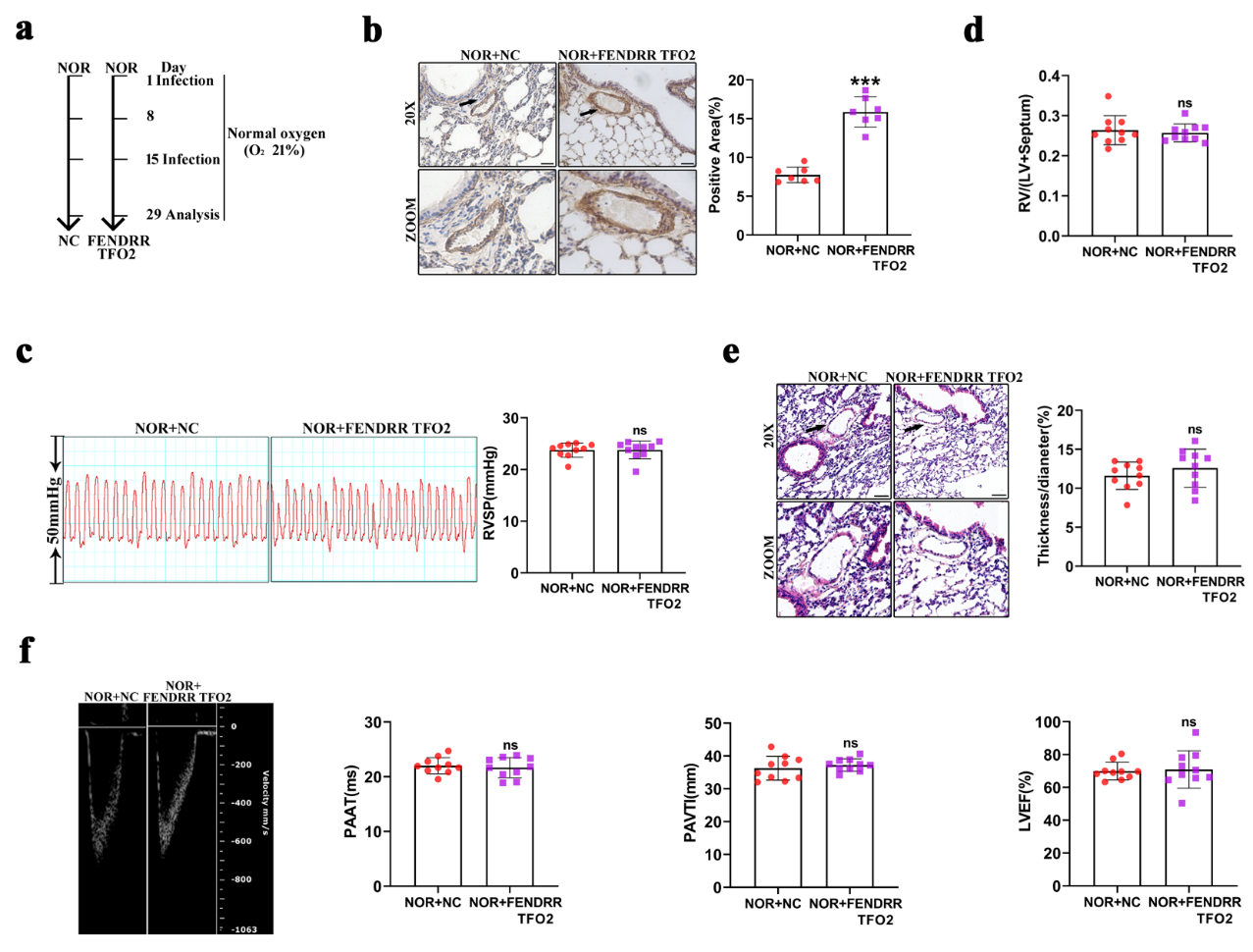


**Additional Fig. S7** Overexpression conserved sequence TFO2 adenovirus of FENDRR in vivo does not affect the development of PH under normoxic conditions. **a** Adenoviruses with NC or TFO2 sequences were used to handle mice via dropwise intranasal instillation every 7 days to mice until 29 days. **b** The functional fragment TFO2 of FENDRR overexpression was verified by in situ hybridization methods (n = 8). Scale bar = 100 μm. **c** Indices of Right ventricular systolic pressure (RVSP) (n = 10). **d** Right ventricle (RV) / (left ventricle (LV) + Septum) weight ratio was calculated (n = 10). **e** HE staining was performed to detect wall thickening (n = 10). **f** Echocardiographic images and indexes (n = 10). All values are presented as the mean ± SD. Statistical analysis was performed with the Student’s t-test. NOR: normoxic; NC: negative control; ns: no significant. ***P < 0.001 compared with NOR + NC.


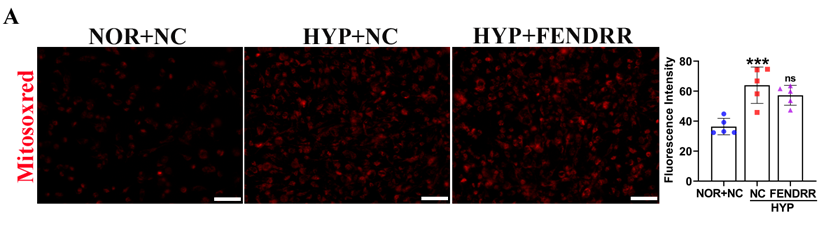


**Additional Fig. S8** **a** Mitochondrial superoxide indicator (Mito-SOX Red) was used to detect the mitochondrial-derived ROS production. Scale bar = 50 μm. NOR: normoxic; HYP: hypoxic; NC: negative control; ns: no significant. ***P < 0.001 compared with NOR + NC.
